# Next‐generation clinical trials: Novel strategies to address the challenge of tumor molecular heterogeneity

**DOI:** 10.1016/j.molonc.2014.09.011

**Published:** 2014-10-18

**Authors:** Daniel V.T. Catenacci

**Affiliations:** ^1^ University of Chicago Medical Center, Department of Medicine, Section of Hematology & Oncology, 5841 S. Maryland Avenue, MC2115, Chicago, IL 60637, USA

**Keywords:** Molecular heterogeneity, Inter-patient heterogeneity, Intra-patient heterogeneity, Next-generation clinical trials, Expansion Platform Designs, PANGEA, Gastric cancer, Esophagus cancer, Gastroesophageal cancer, Esophagogastric cancer

## Abstract

The promise of ‘personalized cancer care’ with therapies toward specific molecular aberrations has potential to improve outcomes. However, there is recognized heterogeneity within any given tumor‐type from patient to patient (inter‐patient heterogeneity), and within an individual (intra‐patient heterogeneity) as demonstrated by molecular evolution through space (primary tumor to metastasis) and time (after therapy). These issues have become hurdles to advancing cancer treatment outcomes with novel molecularly targeted agents. Classic trial design paradigms are challenged by heterogeneity, as they are unable to test targeted therapeutics against low frequency genomic ‘oncogenic driver’ aberrations with adequate power. Usual accrual difficulties to clinical trials are exacerbated by low frequencies of any given molecular driver. To address these challenges, there is need for innovative clinical trial designs and strategies implementing novel diagnostic biomarker technologies to account for inter‐patient molecular diversity and scarce tissue for analysis. Importantly, there is also need for pre‐defined treatment priority algorithms given numerous aberrations commonly observed within any one individual sample. Access to multiple available therapeutic agents simultaneously is crucial. Finally intra‐patient heterogeneity through time may be addressed by serial biomarker assessment at the time of tumor progression. This report discusses various ‘next‐generation’ biomarker‐driven trial designs and their potentials and limitations to tackle these recognized molecular heterogeneity challenges. Regulatory hurdles, with respect to drug and companion diagnostic development and approval, are considered. Focus is on the ‘Expansion Platform Design Types I and II’, the latter demonstrated with a first example, ‘PANGEA: Personalized Anti‐Neoplastics for Gastro‐Esophageal Adenocarcinoma’. Applying integral medium‐throughput genomic and proteomic assays along with a practical biomarker assessment and treatment algorithm, ‘PANGEA’ attempts to address the problem of heterogeneity towards successful implementation of molecularly targeted therapies.

## Highlights

- Tumor molecular heterogeneity is a challenge to the design of clinical trials in Oncology.
- Inter‐patient heterogeneity of low frequency biological events are difficult to study.
- Intra‐patient heterogeneity through space and time require serial molecular profiling.
- We discuss next‐generation clinical trial designs aimed to address heterogeneity.
- Focus is on novel ‘Exploratory’ and ‘Expansion’ Platform clinical trial designs.

## 1 Targeted therapies

Clinical outcomes have significantly improved for most cancers since the introduction of classic cytotoxic agents. Cytotoxic agents can be considered ‘targeted’ in that they inhibit DNA synthesis and the cell division apparatus – the ‘bottleneck’ steps required for cancer to manifest with morbidity and mortality. (Joensuu, 2008) Some stage IV solid tumors, such as testicular cancer, even achieve long term survival with this strategy alone, while in general most advanced solid tumors derive significant palliative benefit for an increased, albeit finite, period of time. Ultimately, solid metastatic tumors develop resistance to cytotoxics, and patients succumb to their illness. A ‘benefit plateau’ has been reached with these cytotoxics. Off‐target ‘collateral damage’ of normal tissues is a well‐recognized potential disadvantage of cytotoxics, necessitating a delicate balance between optimizing tumor control and limiting toxicity.

Genetic aberrations identified within various tumor types, including gene mutation, gene rearrangement, and gene amplification/deletion, led to an understanding of constitutive activation of oncogenes, or loss of function of tumor suppressors, all contributing to a sequential genomic carcinogenesis model. (Fearon and Vogelstein, 1990) The ensuing concept of an ‘oncogenic driver’ and ‘oncogene addiction’ ultimately shifted the course of therapeutics development; (Weinstein and Joe, 2008; Weinstein, 2002; Vogelstein et al., 2013) the era of targeted therapies towards a putative ‘Achilles heel’ was born. (Dancey et al., 2012) In addition to genomic events, abnormalities of protein expression not directly a consequence of a genomic event (ie. abnormally increased protein expression in the absence of mutation, amplification, or translocation of that protein's gene) also received attention for therapeutic potential, as did key signaling ‘nodes’ within critical oncogenic growth and metastasis pathways. (Slamon et al., 1984; Harris et al., 1994; Islam et al., 2013; Bianco et al., 2006) Following this, pharmaceutical agents directly inhibiting the function of a ‘culprit’ protein could be engineered with high selectivity. (Lengauer et al., 2005) Thus, theoretically, these agents would inhibit only cancer cells possessing the dysfunctional (over‐activated or over‐expressed) protein, while sparing normal cells, consequently magnifying the therapeutic window. Attention to essential stromal components of tumors including immune cells, fibroblasts, and endothelial/vascular components also arose. (Devaud et al., 2013; Gimbrone et al., 1972; Kakarla et al., 2012; Bellou et al., 2013; Mueller and Fusenig, 2004; Zitvogel et al., 2006) Over the last decades, the premise of using molecularly targeted agents for targeted patient populations based on tumor/stromal molecular profiles and pathway dependencies gave rise to an array of novel drugs intended to abrogate malignant progression through these ‘specific’ drug–protein interactions. (Griffin, 2001; Mauro et al., 2002; Pegram and Slamon, 2000) Targets now include receptor tyrosine kinases (RTKs) (e.g. HER2, EGFR, MET), intracellular kinases (e.g. PI3K, MEK, AKT), transcription factors (e.g. STAT3), stem cell pathways (SHH/SMO, Notch), immunomodulators (e.g. CTLA4, PD1/PDL1, vaccines), and hormone receptors (e.g. estrogen, progesterone, androgen). Excluding classic cytotoxic inhibition of DNA synthesis and cell division, the main targeted therapy classes include ‘biologics’ (monoclonal antibodies with/without linked cytotoxics known as Antibody‐Drug Conjugates (ADCs)) (Fauvel and Yasri, 2014), ‘small molecules’ such as tyrosine kinase inhibitors (TKIs) (Leary and Johnston, 2007; Faivre et al., 2006), and more recently, specific gene expression silencing by ‘RNA interference’, (Videira et al., 2014; Deng et al., 2014; Yan et al., 2014) each with their own properties, advantages and disadvantages (Table 1).

**Table 1:** General properties of major classes of targeted therapeutics.

Targeted therapy class		Properties	Advantages	Disadvantages
Monoclonal Antibodies	‘Naked’	• Highly specific • IV • ADCC • Long clearance half‐life	• Can be easily combined with cytotoxics • Specific to epitope • Can elicit immune response (ADCC)	• Infusion reactions • Often require concomitant classic cytotoxics for optimal benefit
	Antibody– drug conjugate	• Specific • IV • Targeted delivery of cytotoxic agents	• Can target cytotoxics to tumor cells with potential to increase the therapeutic index	• Ocular/corneal toxicities • Infusion reactions • Less ADCC than naked due to lower numbers of antibody molecules
Small molecules(Kinase inhibitors)		• Usually oral • Often ‘promiscuous’	• Oral administration is appealing • Potential for preemptive inhibition of parallel signaling with one compound	• Compliance • Off‐target effects (promiscuity) lead to toxicity and difficulties in defining accurate predictive biomarkers • Difficult to combine with cytotoxics
RNA interference		• Technical difficulties • siRNA‐based technologies are improving	• Can target currently ‘undruggable’ targets (e.g. KRAS)	• Stability • Off‐target effects • Immunostimulation • Delivery problems

IV, intravenous; ADCC, Antibody‐Dependent Cell‐mediated Cytotoxicity; ‘cytotoxics’, refers to classic chemotherapy directed at inhibiting DNA synthesis and cell division apparatus.

There is now significant evidence supporting the notion that cancer is driven by molecular genetic aberrations. A few well‐known examples following the ‘tumor→genomic driver→matched inhibitor’ paradigm include: ‘CML→ *BCR/ABL* translocation→imatinib’, (Rowley, 1973; Druker et al., 2006; Rowley et al., 1976; Olopade, 2014) ‘Breast/Gastric→*HER2* amplification→trastuzumab’, (Slamon et al., 1987, 2001) ‘GIST→*KIT* mutation→imatinib’, (Demetri et al., 2002) and ‘Melanoma→*BRAF* mutation→dabrafenib/vemurafenib’. (Flaherty et al., 2010; Chapman et al., 2011) Additionally, albeit with generally less dramatic clinical improvements, anti‐angiogenesis within the stromal compartment has demonstrated benefit across solid tumor types. (Bellou et al., 2013; Shojaei, 2012) Inhibition of ‘over‐expressed’ proteins within the tumor – in the absence of genomic aberration of that protein – has less supporting evidence in general, but has shown benefit in randomized phase II settings, such as selection of Met expressing tumors for anti‐MET therapies for gastroesophageal cancer (GEC), (Catenacci et al., 2011a; Iveson et al., 2014) or ATM expression and its potential relevance to PARP inhibition in GEC. (Bang et al., 2013) Most recently, immunomodulation including using immune checkpoint inhibitors have shown benefit in various tumor types, such as tumors expressing PDL1, (Sullivan et al., 2013; Muro et al., 2014) particularly with *concurrent* inflammatory component within the tumor‐bed (Keenan et al., 2013; Le and Jaffee, 2013; June et al., 2014; Maus et al., 2014; Melero et al., 2014; Mellman et al., 2011). Based on these latter proteomic examples, ‘drivers’ or ‘addiction’ need not be considered *only* genomic necessarily; however, the more dramatic improvements in hazard ratios for survival to date are clearly the genomic driver examples (Table 2). (Iveson et al., 2014; Bang et al., 2010; Hecht et al., 2013; Ohtsu et al., 2011; Waddell et al., 2013; Lordick et al., 2013a; Ohtsu et al., 2013; Fuchs et al., 2014; Wilke et al., 2014; Satoh et al., 2014).

**Table 2:**
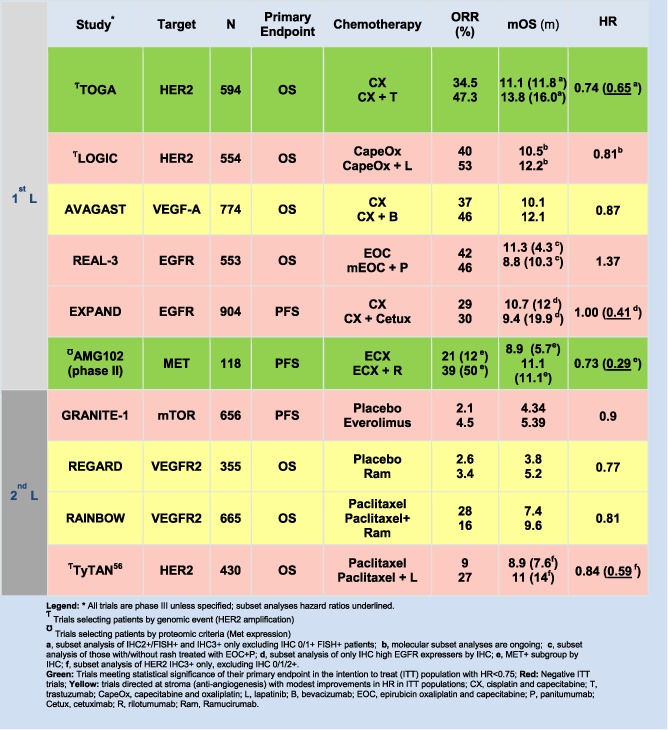
Recent clinical trials with/without biomarker selection for advanced gastroesophageal cancer.

## 2 Inter‐patient tumor molecular heterogeneity: the ‘driver vs wheel’ metaphor

As opposed to the several diverse examples above which targeted sub‐populations for targeted therapy using potentially predictive biomarkers, other evaluations of novel molecularly targeted inhibitors have not been patient‐selective. Among numerous examples (e.g. anti‐EGFR, (Waddell et al., 2013; Lordick et al., 2013a) anti‐mTOR, (Ohtsu et al., 2013) anti‐Hedgehog (Cohen et al., 2013)), clinical trials for GEC based on a ‘one‐size‐fits‐all’ strategy have in general been disappointing. For instance, applying an EGFR inhibitor to the entire GEC population, where genomic activation occurs in only ∼5% of cases (*EGFR* gene amplification) and perhaps in another subset of ∼15–20% of patients with true EGFR ‘over‐expression’ (in the setting of an otherwise normal *EGFR* gene), was not successful (Waddell et al., 2013; Lordick et al., 2013a) (Table 2). Interestingly, the EXPAND trial subset analysis suggested that those patients with tumors within the highest EGFR expressing categories by immunohistochemistry (IHC) appeared to derive survival benefit (HR 0.41) from cetuximab compared to placebo (Lordick et al., 2013b) (Table 2). Other studies since, such as the second line TRANS‐COG erlotinib study, (Petty et al., 2014) have shown similar results in these select patient subsets. (Zhang et al., 2013) However, when lowering the threshold definition of ‘EGFR over‐expressed’, or to the furthest extreme of including all GEC patients, the benefits derived in the small ‘EGFR‐driven’ subsets were seemingly diluted. It is clear that if a similar ‘one‐size‐fits‐all’ strategy was used for anti‐HER2 therapy in GEC, (Bang et al., 2010; Hecht et al., 2013; Satoh et al., 2014) trastuzumab would likely have encountered the same fate as anti‐EGFR agents for this disease (Table 2). This is evidenced within the ToGA trial where subset analyses showed that ‘FISH+, IHC0/1+’ patients derived no benefit from the addition of trastuzumab. (Bang et al., 2010).

The experiences over the last decade with respect to molecular targeted agents saw more negative than positive trials. This has led to the growing acceptance that targeted therapies should be used for targeted patient populations. This is exemplified in Table 2 where GEC trials that have made efforts to select patients in some way (either prospectively or retrospectively) have demonstrated improved outcomes, whereas unselective trials generally have not. It is important to note that within a cancer type, several molecular subsets may be present (Catenacci et al., 2014a; Sehdev and Catenacci, 2013a) (Table 3). This high inter‐patient molecularly heterogeneity from one patient to the next is certainly true for GEC. (Deng et al., 2012; Wang et al., 2011; Holbrook et al., 2011; Zang et al., 2012; Dulak et al., 2012; Co, 2014) In contrast, other cancers, such as CML, are quite homogenous (∼95% *BCL/ABL* translocation), (Druker et al., 2006) partially explaining the success of the ‘one‐size‐fits‐all’ approach initially attempted with imatinib for CML. There is now increased recognition of inter‐patient molecular heterogeneity for most solid tumors. (Bedard et al., 2013; Fedele et al., 2014; Longo, 2012).

**Table 3:**
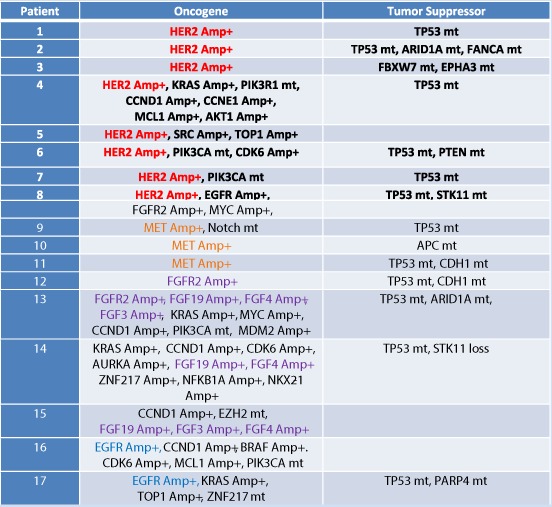
Inter‐patient molecular heterogeneity demonstrated by next‐generation targeted exome sequencingТ.

**Table 3:**
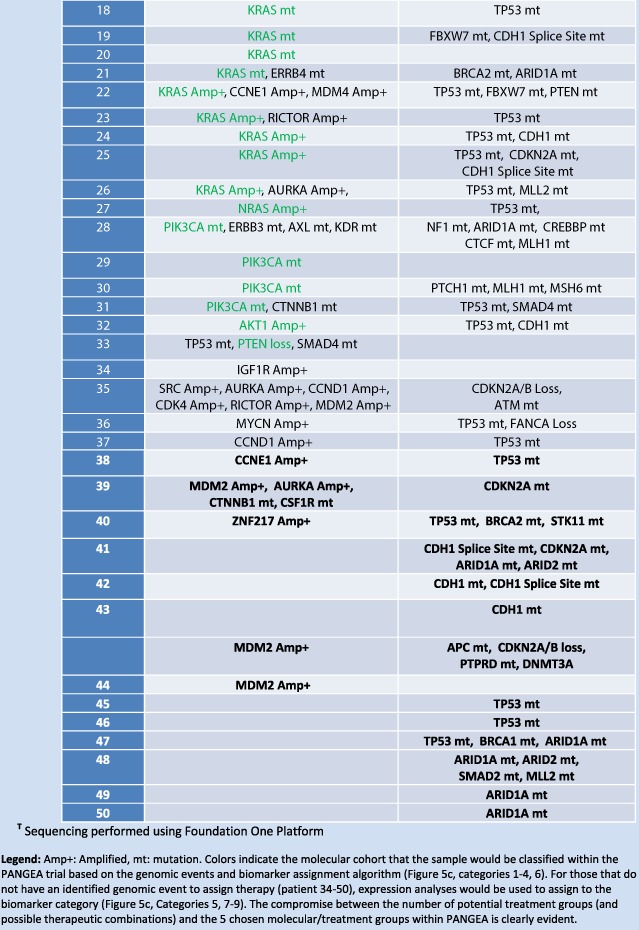
(continued)

**Figure 1:**
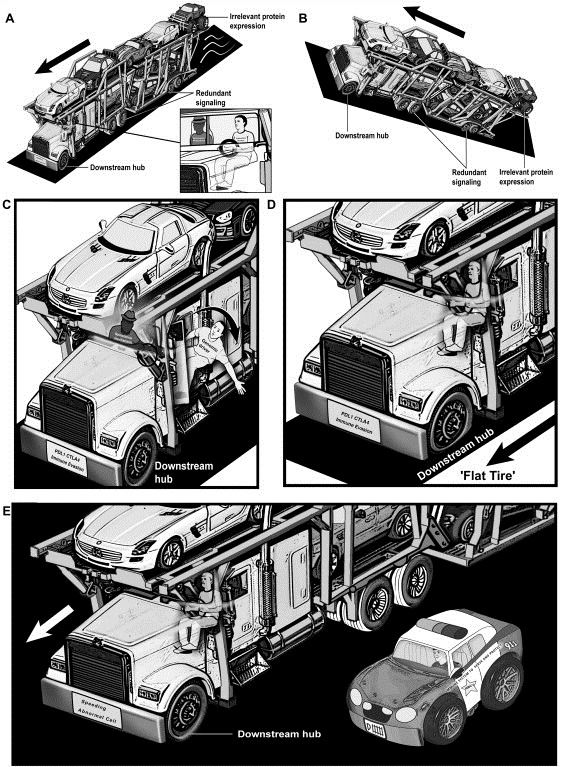
The “run‐away 18‐wheeler truck” metaphor of cancer and current therapeutic strategies. ©Ion Medical Designs, LLC 2014. (A) In the untreated scenario, cancer is like a run‐away truck without brakes (loss of tumor suppressor) quickly and inappropriately accelerating down a hill. (B) In an attempt to slow down the truck (cancer cell), altering the slope (tumor environment) to ‘uphill’ has been employed {eg. anti‐angiogenesis}. (C) Stopping the driver from pushing the gas pedal {targeted inhibition towards the function of the oncogenic genomic driver} may relieve the inappropriate acceleration {eg. trastuzumab for HER2 gene amplification}, if only temporarily until another mechanism (inherent or acquired) to maintain the acceleration stimulus (oncogenic driver) moves to replace it. (D) Although loss of any back wheel (downstream effector) will likely not slow the truck given the presence of numerous wheels (redundant parallel escape signals), some wheels downstream can be critical, like when inducing a flat front tire (critical downstream hub) {eg. inhibition of DNA synthesis: classic cytotoxics; or inhibition of key protein: estrogen/androgen receptor antagonists}. (E) Reversing mechanisms of police (immune) evasion can re‐establish the ability to recognize and eliminate the abnormal ‘speedy truck’ {immunomodulation}. A combination of the strategies in (B–E) may be optimal to slow with significant magnitude and duration.

Continuing with the EGFR example, a ‘driver vs wheel’ metaphor of a ‘run‐away 18‐wheeler truck’ can help to conceptualize the current appreciation of inter‐patient heterogeneity of molecular ‘oncogenic drivers’ (the gas pedal) and loss of tumor suppressors (the brakes) (Figure 1A). When EGFR is the genomic ‘driver’ of a tumor (ie. *EGFR* mutation or *EGFR* amplification; inappropriately ‘pushing the gas pedal’), using targeted inhibition towards that driver generally has resulted in significantly improved clinical outcomes in that patient subset, (Petty et al., 2014; Zhang et al., 2013; Zhou et al., 2011) albeit until development of resistance with consequent progression (Figure 1C). On the other hand, in settings when EGFR is ‘over‐expressed’ without genomic activation, or even less impressively merely ‘expressed’, similar to any of hundreds to thousands of other proteins in a tumor, EGFR may only be one of many wheels (downstream effectors) on the truck (cancer cell) (Figure 1A). Therefore, it is not critical and is easily expendable if neutralized, with other wheels (parallel escape signaling) taking up the slack. (Waddell et al., 2013; Lordick et al., 2013a).

However, certain wheels may be more important than other downstream wheels, acting as critical downstream ‘hubs’ (ie. a front wheel flat tire versus one in the back will slow the truck more effectively, Figure 1D). Targeted inhibition of critical non‐genomically‐activated downstream ‘nodes’ (e.g. ER/PR antagonists for ER/PR + breast, CD20 antagonists for Lymphoma, HGF antagonists for MET+ GEC (awaiting phase III validation), and even classic cytotoxics inhibiting the bottleneck cell division apparatus) are examples that may represent this approach (Figure 1D). It is essential to recognize that a gene/protein can be a driver in some patients within a given tumor type (*EGFR* amplification) yet more commonly only a minor wheel (or even a bystander) in the majority of patients with that same tumor type. Table 3 reveals the vast inter‐patient heterogeneity observed with respect to genomic driver events and suppressor loss across 50 GEC patients. (Catenacci et al., 2014a; Sehdev and Catenacci, 2013a) Therefore, clinical trials that have not made this distinction have been either negative, or do not observe as substantial a benefit as those seen when selecting subsets with a true oncogenic ‘driver’ or a dependent downstream hub. Targeting a downstream hub within the cancer cell may be the best or only option in some cases, where genomic events are currently not directly actionable (e.g. *KRAS* mutant/amplified cancers (Catenacci et al., 2013; Baines et al., 2011)). Synthetic lethality may circumvent loss of tumor suppressors (the brakes) and/or oncogenic activating events that are not actionable, (Kaelin, 2005) and could be considered a critical hub. In contrast, inhibitory strategies directed at the tumor stroma (e.g. anti‐angiogenesis) alter the environment (Figure 1B), and in the metaphor can be thought of as forcing the truck to go uphill, thus slowing it down. Anti‐angiogenesis has no predictive biomarker identified to date, which may be due to a universal benefit across unselected patients (that is marginal) (Table 2, yellow). Finally, immunomodulatory agents (e.g. anti‐PD1/PDL1, anti‐CTLA4, vaccines, adoptive transfer of tumor infiltrating lymphocytes) are an orthogonal approach, that may not be dependent on genomic drivers, that may re‐establish the capability of the host immune system (police in the metaphor) to recognize, ‘catch’, and remove tumor cells in appropriately selected patients (Muro et al., 2014; Mellman et al., 2011) (Figure 1E).

In short, there are several instances where more substantial clinical benefit was observed when using targeted therapies for targeted populations (directed at the genomic driver or a critical downstream hub) than when using targeted therapies in untargeted, or the whole of, populations. However, a combination of one or more of these strategies (targeting the genomic driver(s), downstream hub(s), and/or the orthogonal strategies towards tumor stroma with anti‐angiogenesis and/or immunomodulation) may provide an optimal approach to ‘slowing down or stopping the truck’.

Working under the premise of ‘driver’ biology with matched targeted therapeutics, inter‐patient heterogeneity of activated oncogenes has obvious implications on our targeted treatment strategies *and* clinical trial designs.

## 3 Intra‐patient tumor molecular heterogeneity

**Figure 2:**
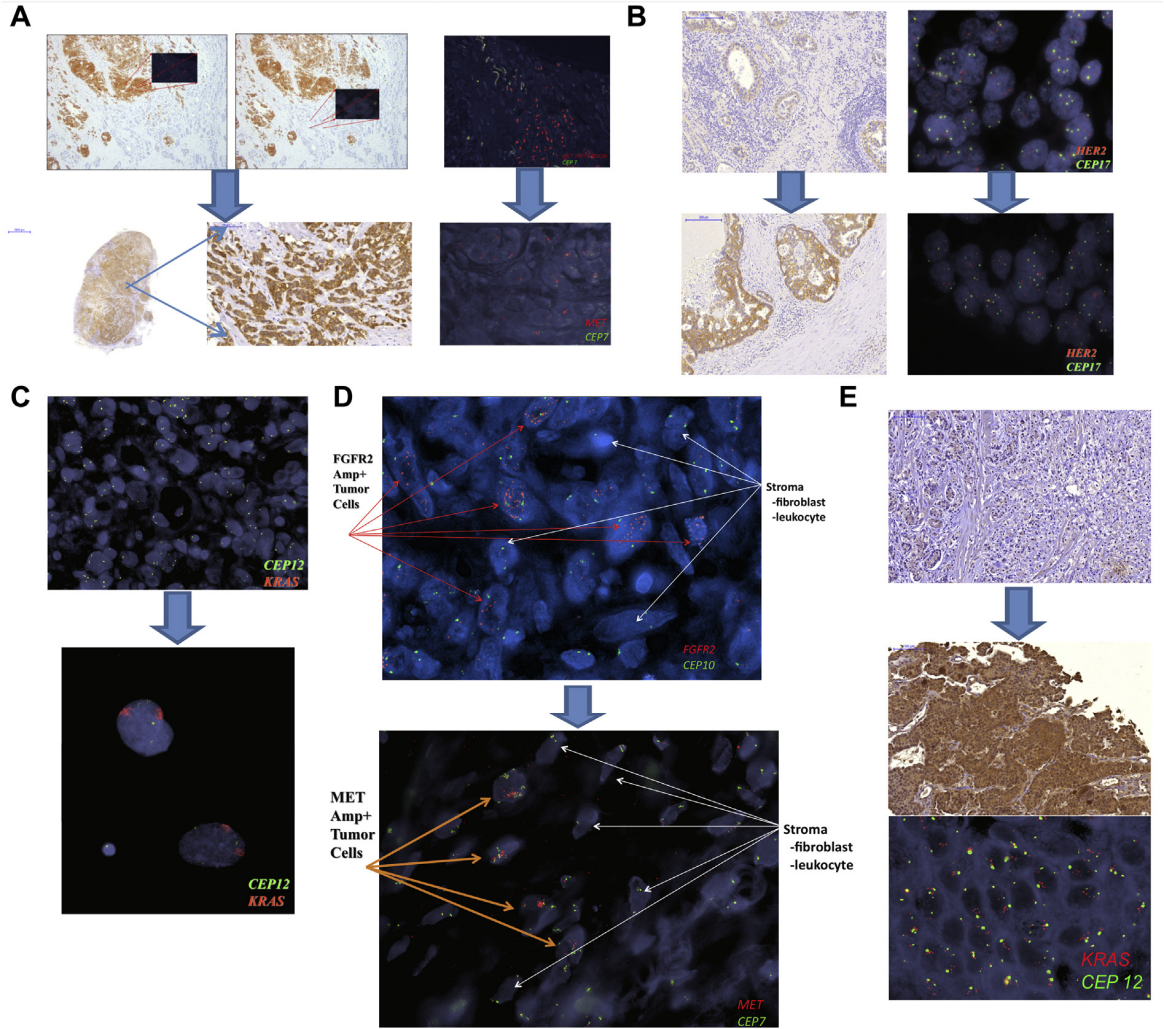
Intra‐patient tumor molecular evolution through space and/or time. (A) Intra‐patient heterogeneity ‘through space’ of Met by IHC (left) and MET gene copy by FISH (right) within the primary tumor (upper panel) to metastatic lymph node (lower panel. (Catenacci et al., 2014a) (B) Intra‐patient heterogeneity ‘through space’ of Her2 by IHC (left) and HER2 gene copy by FISH (right) from primary tumor (upper panel) to metastatic lymph node (lower panel). (Catenacci et al., 2014a) (C) Intra‐patient heterogeneity ‘through space’ of KRAS gene copy by FISH in primary tumor (upper panel) to metastatic peritoneal ascites (lower panel). (Catenacci et al., 2013) (D) Intra‐patient heterogeneity ‘through space and time’ of tumor cells and stromal elements within the primary tumor at diagnosis (upper panel) and metastatic peritoneal carcinomatosis implant after cisplatin/5FU chemotherapy (lower panel). FGFR2 is gene amplified only in the primary tumor, and MET is gene amplified only in the metastatic deposit. (Catenacci et al., 2014b) (E) Intra‐patient heterogeneity ‘through space and time’ of KRAS gene copy and expression prior to anti‐Met antibody therapy (upper panel, normal gene copy) and after (lower panel, gene amplified) suggesting a mechanism of resistance. (Catenacci et al., 2011a, 2014a; Catenacci et al., 2013).

Despite the clinical gains realized following the matched “oncogenic driver→targeted therapeutic” strategy, other hurdles have prevented more substantial benefit. These hurdles include intra‐patient molecular heterogeneity through space (within the patient), and over time (before and after therapy) – both of which have been reported across solid tumors. (Yap et al., 2012; Gerlinger et al., 2012) Examples of intra‐patient tumor heterogeneity through space, either within the primary tumor, or from primary tumor to an involved lymph node and/or distant metastatic site are shown in Figure 2A–C. This is not a new concept. (Nowell, 1976) In some series, rates of molecular evolution for a given biomarker through space at a given ‘snap‐shot’ in time are as high as 10–15%. (Gerlinger et al., 2012; Swanton, 2012) Other studies have minimized the rate and significance of this evolution, usually when typically only evaluating a few select genes/proteins in the study. (Vakiani et al., 2012; Vignot et al., 2013) This may be tumor specific. However, the ultimate evidence of tumor evolution through space was demonstrated using high‐throughput next‐generation genomic sequencing (NGS), offering a remarkable illustration of Darwinian evolution and natural selection at the cellular level in renal tumors. (Gerlinger et al., 2012; Hull, 2005; Navin et al., 2010; Navin and Hicks, 2010) Clearly, misclassification of a tumor as ‘HER2 negative’ based on the primary site may have implications on outcomes for that patient if the metastatic site had evolved to acquire *HER2* amplification (Figure 2B). (Seol et al., 2012; Arena et al., 2013; Lee et al., 2013a; Yoon et al., 2012) Further realization of continued tumor evolution and adaptation through time with consequent therapeutic resistance, has been extensively described pre‐clinically, (Catenacci et al., 2011a; Turke et al., 2010; Corso and Giordano, 2013; Cepero et al., 2010; Engelman et al., 2007) and exemplified clinically via pre/post therapy tumor biopsies (Figure 2D and E). Building on the “Driver‐Wheel” metaphor, the tumor mass is composed of large populations of cancer cells (a populations of trucks). Developed resistance and disease progression on therapy may be due to i) inherent concomitant genomic resistance mechanisms within the majority of cancer cells (Figure 1C) rendering immediate resistance, ii) inherent reactive or ‘adaptive’ resistance mechanisms within the tumor/stroma DNA blueprint, in the absence of other genomic events, leading to a responsive/adaptive signaling pathway ‘rewiring’ rendering immediate or eventual resistance, (Wilson et al., 2012) and/or iii) clonal selection of the cancer cell sub‐populations (certain trucks) possessing additional genomic drivers with/without the originally identified genomic event, rendering eventual resistance. In this third scenario although some cells (trucks) are sensitive to (and eliminated by) the therapy, those possessing genomic events that provide mechanisms of resistance will persist. Given this, trials matching a targeted agent towards a targeted genomic event may successfully derive benefit, with varying duration depending on the time to selection and full expansion of subclonal populations (resistant trucks) leading to eventual drug failure.

Working under the premise of ‘driver’ biology with matched targeted therapeutics, even if appropriately matched at the onset, molecular evolution and selection through space and time also has apparent implications on our targeted treatment strategies *and* clinical trial designs.

## 4 The challenge of molecular heterogeneity in the design of clinical trials

### 4.1 Inter‐patient tumor molecular heterogeneity

**Figure 3:**
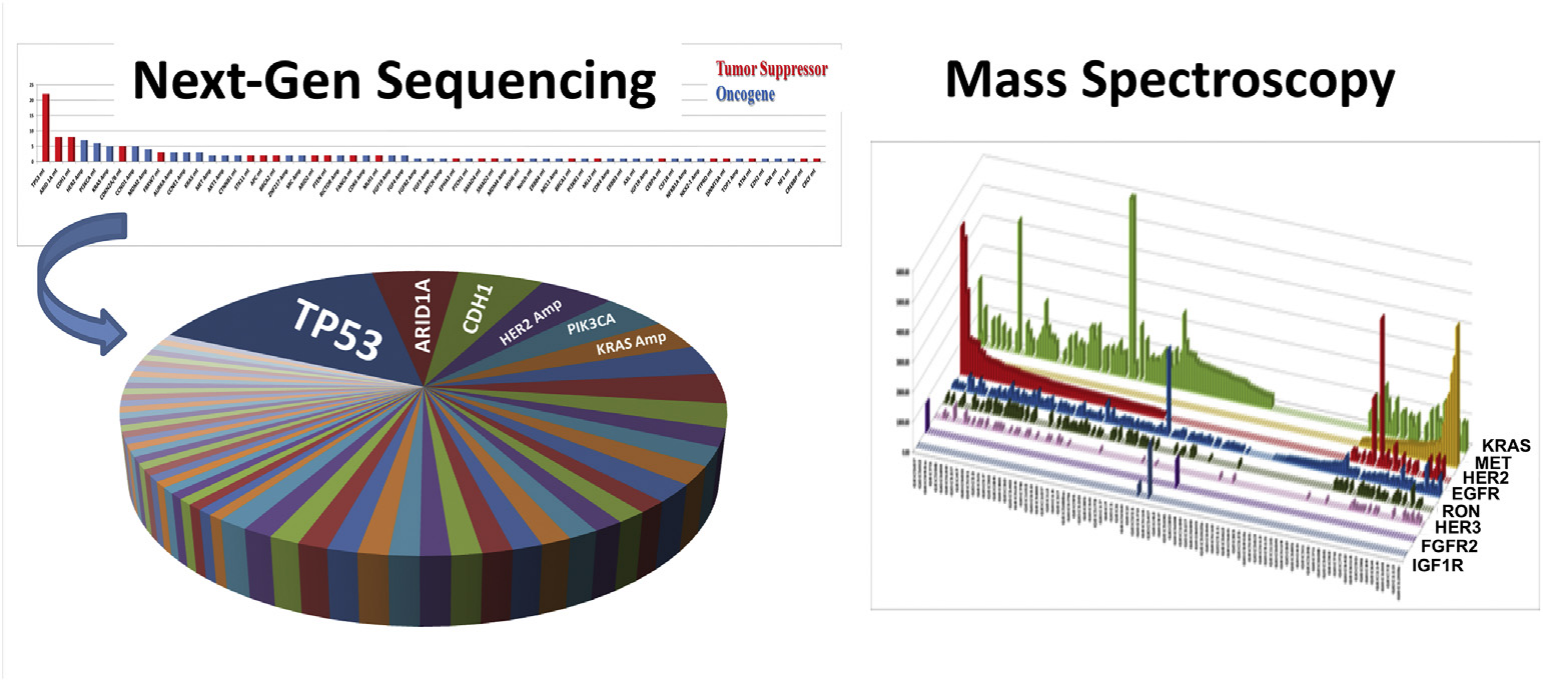
Inter–patient tumor molecular heterogeneity. (Left panel) Genomic profiling using a ∼240 gene next‐generation sequencing (NGS) platform of a cohort of 50 stage IV GEC samples (upper panel) revealing few high frequency events (peak) and numerous low frequency events (tail); pie chart revealing profound inter‐patient molecular heterogeneity (see Table 3). (Catenacci et al., 2014a) (Right panel) Proteomic expression profiling of 100 GEC samples using multi‐plex (8 peptides shown) selected reaction monitoring (SRM) mass spectrometry (MS) revealing clear inter‐patient heterogeneity. (Catenacci et al., 2014a,b; Hembrough et al., 2012).

The ToGA trial evaluated trastuzumab for ‘HER2 positive’ GEC, (Bang et al., 2010) and screened 3803 patients internationally to obtain 810 eligible patients, of which 594 were otherwise eligible for randomization (Table 2). To be eligible, ‘HER2 positive’ was defined as a ‘FISH+’ ratio ≥2 with any IHC score (0‐3+), *or* IHC3+ with ‘FISH‐‘; ‘IHC2+/FISH‐‘ patients were ineligible. After excluding ineligible patients by these biomarker screening criteria, as well as those not meeting other trial entry criteria, only 15.6% of all‐comers with stage IV GEC were eligible for therapy. Based on preplanned subset analyses, ‘HER2 positivity’ is now clinically defined with a more stringent threshold than even ToGA initially used for screening: (IHC2+/FISH+, IHC3+/anyFISH), which would exclude the 131 patient tumors with FISH+/IHC0‐1+ scores who appeared to derive no benefit from the addition of trastuzumab. That leaves 463 patients from the original 3803 screened patients (12%), or 57% of the initially identified ‘HER2+ patients’ in the trial. By acknowledging the disappointments of applying targeted therapies in a ‘one‐size‐fits‐all’ strategy, the ToGA trial illustrates the ongoing challenge when attempting to select patients for targeted therapies. This includes the extremely high numbers of patients required to screen when attempting to apply classic clinical trial designs, with frequentist statistical methods, (Simon and Maitournam, 2004) to subsets within a very molecularly heterogeneous disease such as GEC. Worse, the example of HER2, entailing ∼10–15% of GEC, is one of the larger ‘slices of the pie’ (Figure 3, Table 3). The accrual numbers that were required for the ToGA trial demonstrates how profound inter‐patient molecular heterogeneity is challenging the application of novel targeted agents for specific sub‐populations using traditional clinical trial designs. Selecting patients with *MET* amplified tumors at ∼4% incidence within GEC for anti‐MET therapy, (Smolen et al., 2011, 2014, 2006) which is based on sound preclinical and clinical evidence, is an even more difficult challenge than the *HER2* ToGA example. Such a phase III trial would require >15000 total GEC patients with stage IV disease to be screened to accomplish a ‘*MET* amplified‘ phase III selection trial. When also considering that there are several redundant drugs adopting the same strategy for this limited patient cohort, a large randomized phase III trial is seemingly impossible (as is even a randomized phase IIb). Importantly, there is increasing recognition of multiple rare molecular subsets (including both genomic events or proteomic expression ‘cut‐offs’ that are considered biologically vital) within solid tumors. Therefore, selection strategies within biomarker‐driven trials using ‘à la carte’ low throughput companion diagnostic assays, such as IHC, PCR or FISH, result in sizeable screening delays. (Stricker et al., 2011) With each diagnostic test having its own central site, serial eligibility screening for each individual trial is required – all while the patient awaits without therapy. Patient drop‐out due to wait‐time is high in this setting, unfortunately. Moreover, in the stage IV setting, the tumor sample is most often from a small tissue biopsy that is ultimately exhausted via repeated serial analyses, precluding further screening for trial eligibility without a repeat tissue biopsy. Further, the odds of qualifying for a given trial are low given the relative infrequency of each aberration. Crucially, multiple *concurrent* events (genomic and proteomic) within a given tumor biopsy, often not fully appreciated when presented as pie charts or ‘high‐peak long‐tail charts’, further complicate treatment stratification (Table 3). (Catenacci et al., 2014a) For instance, focusing solely on *genomic* aberrations without considering proteomic profiling, one tumor sample can range from having 0–18 identified ‘actionable’ events on medium‐throughput NGS platforms (∼250‐300 genes). (Catenacci et al., 2014a; Sehdev and Catenacci, 2013b; Frampton et al., 2013) Which of the multiple genomic events should be targeted? How to navigate the infinite possible drug‐combinations without established phase I data? What if the drug is not yet available commercially and no trial immediately available? How do we test the hypothesis of each actionable molecular event and ‘matched’ drug with statistical power to rule out effect from random variation (which to the extreme, ultimately approaches an ‘N‐of‐1’ trial)? (Parmigiani et al., 2009) The number of patients required, the limited amount of tissue available, and the length of time to results acquisition, along with the dilemma of multiple ‘actionable’ events in a given sample, all highlight serious challenges currently imposed by inter‐patient tumor molecular heterogeneity.

### 4.2 Intra‐patient tumor molecular heterogeneity

In addition to the risk of misclassifying tumors that have evolved through space when profiling primary disease site biopsies (Figure 2A–C), there are many other obstacles to successfully implementing targeted therapies for patients arising from intra‐patient molecular heterogeneity. It is not uncommon that ‘outdated’ biopsies or previous curative intent surgical resections of primary tumors, often dating months to years earlier and with multiple intermediate lines of therapy, are used to molecularly profile a tumor and dictate treatment stratification in the present. The likelihood of tumor evolution through both space *and* time in this common scenario is considerable (Figure 2). (Yap et al., 2012; Gerlinger et al., 2012) Although the challenge of imposing a biopsy in ‘real time’ had been prohibitive over the last decade, (Dowlati et al., 2001) recently, this is becoming more accepted and even *required* for biomarker stratified trial enrollment. (Poplin et al., 2013) However, systematic acquisition of post‐treatment progression biopsies to directly assess tumor evolution pre/post that particular therapy, often branded as “research” or “optional”, (Lee et al., 2013b; Overman et al., 2013) have been largely unsuccessful with physician reluctance and high patient drop‐out. This has led to lost opportunity to systematically evaluate reasons for immediate drug failure, or eventual resistance in the event of an initial response. Perhaps most importantly, there is lost opportunity to acquire the knowledge how to guide therapy for that patient going forward based on the molecular changes observed.

## 5 Biomarker‐focused trials, heterogeneity, and next‐generation clinical trials

**Figure 4:**
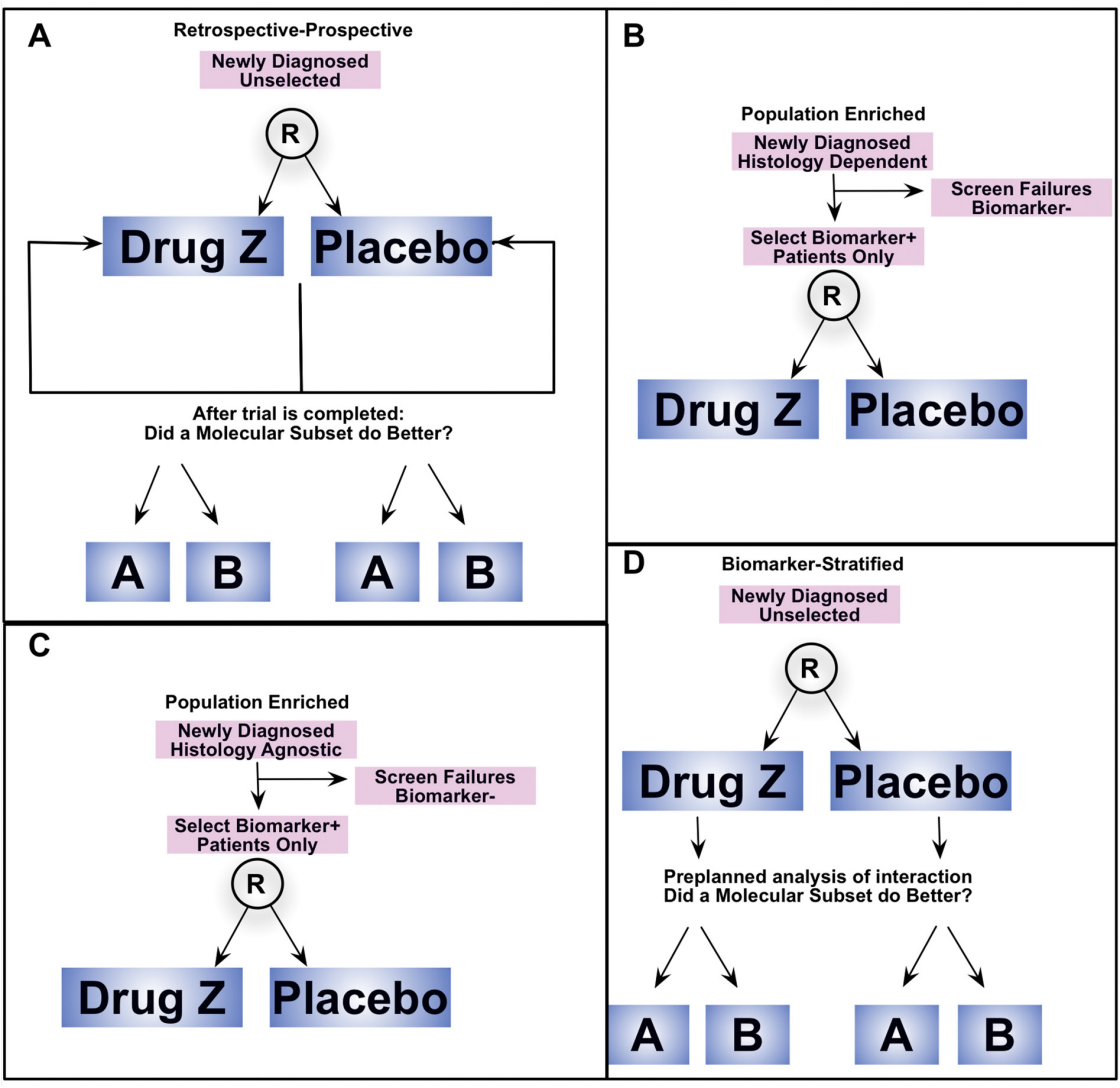
Classic biomarker‐focused clinical trial designs. (A) Retrospective‐Prospective. (B) Population Enriched, Histology Dependent. (C) Population Enriched, Histology Independent. (D) Biomarker Stratified.

With increased recognition of the relevance of molecular ‘driver‐biology’ along with the obstacles posed from inter‐ and intra‐patient heterogeneity, various biomarker‐focused trial designs have been utilized to evaluate a biomarker's ability to predict treatment response (Table 4, Figure 4). A biomarker's predictability of benefit can be assessed within two broad categories – retrospective (but prospectively evaluated) or prospectively planned studies. Retrospective‐prospective approaches have been a mainstay approach, for example with *KRAS* (De Roock et al., 2010; Catenacci et al., 2011c) (and now *NRAS* (Douillard et al., 2013, 2013)) mutations demonstrating lack of benefit of anti‐EGFR monoclonal antibody therapies in colorectal cancer (Figure 4A). Notably this design requires sufficient numbers of tumor samples from the original trial to be assessed in order to limit selection bias. (Simon et al., 2009) It is also limited to higher incidence biomarker subgroups (*KRAS* mutation ∼40% of colorectal cancers) such that there is adequate statistical power to identify a differential benefit based on the biomarker.

**Table 4:** Properties of various biomarker‐driven clinical trial designs.

Biomarker trial design	Advantages	Disadvantages
Retrospective‐prospective	• Utilize prior trials retrospectively (e.g. RAS for colon cancer therapy) • Useful for exploratory biomarkers not known at time of trial execution • Expedient results for biomarkers	• Tissue availability often not adequate in unplanned trials leading to selection bias • Multiple ‘à la carte’ biomarker assays exhaust limited tissue samples – unable to evaluate true inter‐patient heterogeneity • One molecular ‘snap‐shot’, often not immediately prior to treatment • Requires large numbers of patients for adequate power • Requires high frequency of the biomarker for adequate power

Classic population enriched	• Prospectively select for a biomarker	• High screen failure rates for lower incidence events = wasted tissue/patients • Multiple ‘à la carte’ biomarker assays exhaust limited tissue samples – unable to evaluate true inter‐patient heterogeneity • High patient drop out while awaiting multiple tandem biomarker screenings

• Histology Dependent	• Tumor‐specific outcomes clear path to FDA approval if event is relatively frequent (e.g. HER2 breast, GEC)	• Difficult to accrue for rare events for large phase III trial • Difficult for FDA approval if rare event • A trade‐off of added patient heterogeneity (ethnicity/geography) to enhance accrual via large international trials

• Histology Independent	• Enrich only for a rare ‘driver’ event without attention to tumor site of origin • Enhanced accrual for that aberration	• Heterogenous tumor types, treatments (cytotoxics), and outcomes • Still difficult to accrue very rare events • Difficult to reach statistical significance and path to FDA approval

Biomarker stratified	• Ideal to prove specificity of benefit only to those with biomarker present by including both patients with and without the biomarker • Easier to accrue to given no selection at enrollment • Adaptive randomization can decrease drug exposure of biomarker‐negative patients	• Large sample sizes needed to test the biomarker interaction • Biomarker‐negative patients treated that are hypothesized to not benefit • Wasteful of patients having tumors with other biomarkers that could be better treated with a more appropriate inhibitor • Off‐target effects for ‘promiscuous’ inhibitors will bias the biomarker status interaction towards the null

Exploratory platforme.g. ‘BATTLE’, ‘I‐SPY’	• Ideal to assist in identifying the best molecular subset for a drug, if this is previously unknown, in phase I‐IIb trials • Can address inter‐patient molecular heterogeneity with multiple drug ‘bins’, with efficient prospective biomarker testing • Adaptive statistical design to confirm early efficacy signals in later stages of the trial • Can theoretically spin‐off ‘winning combinations’ of new biomarker‐drug matches to confirm in a larger phase III trial, with clear path to FDA approval • Dynamic and iterative – add/remove drugs	• Requires very high numbers of patients for adequate power from start to FDA approval of a drug • Difficult to accrue for follow up large phase III trials if biomarker is rare, as in ‘Population Enriched’ cohorts above • Initially not truly personalized (randomized to each drug bin) for many patients • Not ideal if a strong preclinical or clinical association between a biomarker and drug is already established (e.g. trastuzumab and HER2 amplification) • Ideally, biomarker subsets are chosen beforehand, so they must be known, but design is flexible to add newly identified molecular subsets • Requires multiple drug cohorts and therefore extensive coordination between various pharmaceutical collaborations
Expansion platform	• Umbrella biomarker enrichment that addresses inter‐patient heterogeneity with efficient molecular profiling and treatment assignment • Ideal if biomarker‐drug association is already established	• Assumes drug is only useful for a certain biomarker, or at least best suited for that biomarker

• Type IA: Global and compartmentalized Histology dependent: e.g. ‘FOCUS‐4’	• Can test defined biomarker subsets within a cancer with a drug (or drug combination) thought best matched to that biomarker cohort in an organized global approach for that specific tumor type • Each biomarker cohort is run as its own phase IIa or b trial (compartmentalized), likely with a separate principal investigator • Dynamic and iterative – can add/remove cohorts and matched drugs in real‐time • Treatment has (or should have) a prioritized scheme, acknowledging multiple aberrations in a given tumor	• Requires top‐down coordination and centralization (feasible in centralized health care systems like the United Kingdom or in large cooperative groups/NCI‐CTEP or large pharmaceutical companies with many drugs) • Requires very high numbers of patients as each cohort is considered its own separate trial with individual statistical endpoints – infrequent biomarker incidence is not adequately addressed, particularly for less common tumor types • Arguably, still requires a confirmatory phase III trial for each of the cohorts that have positive signals at the randomized phase IIb setting, requiring even more patients in the population enrichment phase III design • Treatment algorithm can be considered arbitrary and may not have consensus amongst investigators

• Type IB: Global and compartmentalized Histology agnostic: eg. ‘NCI‐MATCH’, & ‘Signature’	• Can test defined biomarker subsets in any tumor type with a drug (or drug combination) thought best matched to that biomarker cohort in an organized global approach for that specific tumor type • Each biomarker cohort is run as its own phase IIa or b trial (compartmentalized) with a separate principal investigator • Dynamic and iterative – can add/remove cohorts and matched drugs • Wide participation (including private oncology clinics), central IRB and screening can screen large numbers of patients	• Requires top‐down coordination and centralization (feasible in centralized health care systems like the United Kingdom or in large cooperative groups/NCI‐CTEP or large pharmaceutical companies with many drugs) • Requires very high numbers of patients as each cohort is considered its own separate trial with individual statistical endpoints – i.e. infrequent biomarker incidence is not specifically addressed, particularly for less common tumor types • There is a trend of using the weaker primary endpoint of response rate in phase IIa trials (Signature) • Arguably, still requires a confirmatory phase III trial for each of the cohorts that have positive signals at the randomized phase II setting, requiring even more patients in the population enrichment phase III design (and decision whether or not to select for specific histology) • There is not a treatment algorithm and therefore tumors with multiple mutations are randomly selected to one of many possible biomarker groups • Assumes aberrations are identical across differing tumor histologies, which is not always confirmed (e.g. BRAF mt in melanoma vs colon)

• Type IIA: Grass‐Roots and Holistic eg. ‘PANGEA’	• A holistic approach to a specific cancer type within one trial, drastically reducing the total number of patients required • Treating one tumor type with tumor‐specific cytotoxics, strategies, and diagnostics • All patients are eligible, given relegation tiers • One center can run pilot phase IIa trials • Randomized phase IIb iterations can be accomplished with small collaborative groups • A number of ongoing trials can be done at various centers, testing various aspects of the personalized approach (Table 5) • Positive phase IIb trials can move to the phase III setting to test the ‘Holistic’ approach OR positive cohorts within the phase IIb can spin‐off to their own phase III trial	• Multiple treatment arms within one trial, which is challenging to negotiate different companion diagnostics and drugs for each identified biomarker subset • Treatment algorithm can be considered arbitrary and may not have consensus amongst investigators, but given the low numbers required, the algorithm can be tested quickly with one/few sites, while other algorithms can be tested simultaneously within separate parallel Type IIA trials performed at other sites. • Despite rationale for such a design, regulatory structure and FDA approval of a trial encompassing multiple molecular subsets each treated with a matched therapy towards one common statistical endpoint is uncertain currently, deterring Pharma and Companion Diagnostics company participation

• Type IIB: Grass‐Roots and Holistic *With ‘Biologic Beyond Progression’ (BBP) e.g. PANGEA‐BBP	• The only biomarker‐driven trial to address intra‐patient tumor heterogeneity over time due to resistance in sequential fashion • Sequential nature of BBP allows for less confounding of post‐protocol therapies for overall survival endpoint, and also less selection bias at second or third line setting • A randomized phase IIb can evaluate overall survival of a ‘personalized holistic approach’ compared to standard therapy • Those positive phase IIb trials can move to the phase III setting to test the ‘Holistic’ approach OR positive cohorts within the phase IIb can spin‐off to their own phase III	• Multiple biopsies are required, a potential deterrent for some patients/physicians • Treatment algorithm can be considered arbitrary and may not have consensus amongst investigators, but given the low numbers required, the algorithm can be tested quickly with one/few sites, while other algorithms can be tested simultaneously within separate parallel Type IIA trials performed at other sites • Despite rationale for such a design, regulatory structure and FDA approval of a trial encompassing multiple molecular subsets each treated with a matched therapy towards one common statistical endpoint is currently uncertain, deterring Pharma and Companion Diagnostics company participation

On the other hand, there are various types of prospective biomarker‐driven trials (Table 4). (Mandrekar and Sargent, 2011; Sargent et al., 2005; Freidlin et al., 2010; Lai et al., 2012) Prospective population enriched integral designs, such as the ToGA trial mentioned earlier (Bang et al., 2010), predefine an eligible population by the presence of the biomarker (e.g. *HER2* amplification) and test the targeted agent (e.g. trastuzumab) only in that population (Figure 4B). This design inherently requires preclinical validation of this strategy with respect to both the companion diagnostic in order to accurately identify the biomarker, and the drug's unique benefit only in tumors with this integral biomarker. (Dancey et al., 2010) A more recent type of population enriched design ignores traditional tumor histologic origin and classification (eg, gastric, lung etc.) by enriching for a certain biomarker, such as *PIK3CA* mutation or *MET* amplification, irrespective of tumor histology (Figure 4C). In attempt to overcome the rarity of the aberration and enrich the cohort in early phase trials using this ‘histology‐agnostic’ strategy, new sets of hurdles arise. Interpreting treatment outcomes in the context of heterogeneous tumor‐specific outcomes is challenging, particularly without a placebo and histology‐stratified control. Moreover, differing sub‐specialty oncologists, within current academic clinical trial infrastructure, enrolling to one such trial may be difficult to coordinate. Finally, differing chemotherapy backbones for different cancer types (if combining cytotoxics with the targeted agent), and ultimately lacking a clear path towards FDA approval (discussed below, Figure 8) are all factors challenging this design approach.

Biomarker stratified (or marker‐by‐treatment interaction) integrated designs aim to evaluate both a new treatment and a biomarker within the same trial (Figure 4D). (Freidlin and Korn, 2014) These randomized phase IIb trials enroll all‐comers within the disease‐type, with a planned biomarker subset analysis. If the phase IIb shows a significant interaction of effect in patients with/without presence of the biomarker, then integral selection of only patients with the biomarker for the confirmatory phase III trial would follow. A recent example of this approach includes the rilotumumab (AMG‐102, anti‐HGF) monoclonal antibody for GEC, (Iveson et al., 2014) which was non‐selective in the randomized phase IIb, but based on the interaction of MET expression status, the ongoing phase III (NCT01697072, NCT02137343) currently selects only MET‐positive tumors – as determined by IHC (Table 2). Larger sample sizes are needed statistically in the phase IIb biomarker stratified design in order to test the interaction, particularly if the biomarker is of low incidence, and a number of biomarker‐negative patients are required to be enrolled, who are theoretically not expected to gain benefit based on the presumed link between the ‘driver biomarker’ and targeted therapy. However, adaptive randomization can limit drug exposure in biomarker‐negative patients. (Wang et al., 2012) In this design, clearly, the specificity of the inhibitor is important, as promiscuity of the drug, such as observed with numerous kinase inhibitors, (Karaman et al., 2008) can confuse matters when responses due to off‐target drug effect occurs (Table 1). For instance, an observed response in an *ALK* wild‐type tumor having an unidentified *MET* amplification in a patient enrolled on a biomarker stratified lung cancer trial that is evaluating crizotinib therapy and the interaction of *ALK*‐translocated versus *ALK* wild‐type tumors would bias the results towards the null (a response in the biomarker‐negative group due to *MET* amplification).

Although each of these ‘classic’ biomarker trial designs above have potential advantages and disadvantages (Table 4), perhaps the most notable disadvantage is the ‘à la carte’ nature of both the companion diagnostic and the ‘biomarker→drug matching’, which neglects the numerous concerns raised earlier regarding immense inter‐patient and intra‐patient molecular heterogeneity.

### 5.1 ‘Umbrella’ or ‘platform’ next‐generation clinical trials

In recognition of the numerous challenges described above by both inter‐ and intra‐patient tumor molecular heterogeneity, newer trial designs have emerged referred to herein as ‘Next‐Generation Clinical Trials’. The designs discussed below each can evaluate novel targeted agents as monotherapy or in combination with standard cytotoxics and/or targeted agents, and most importantly, they can also assess ‘novel–novel’ combination approaches/strategies and efficient ways to select and test these combinations (Yap et al., 2013).

#### 5.1.1 Exploratory platform (e.g. ‘BATTLE’, ‘I‐SPY’)

**Figure 5:**
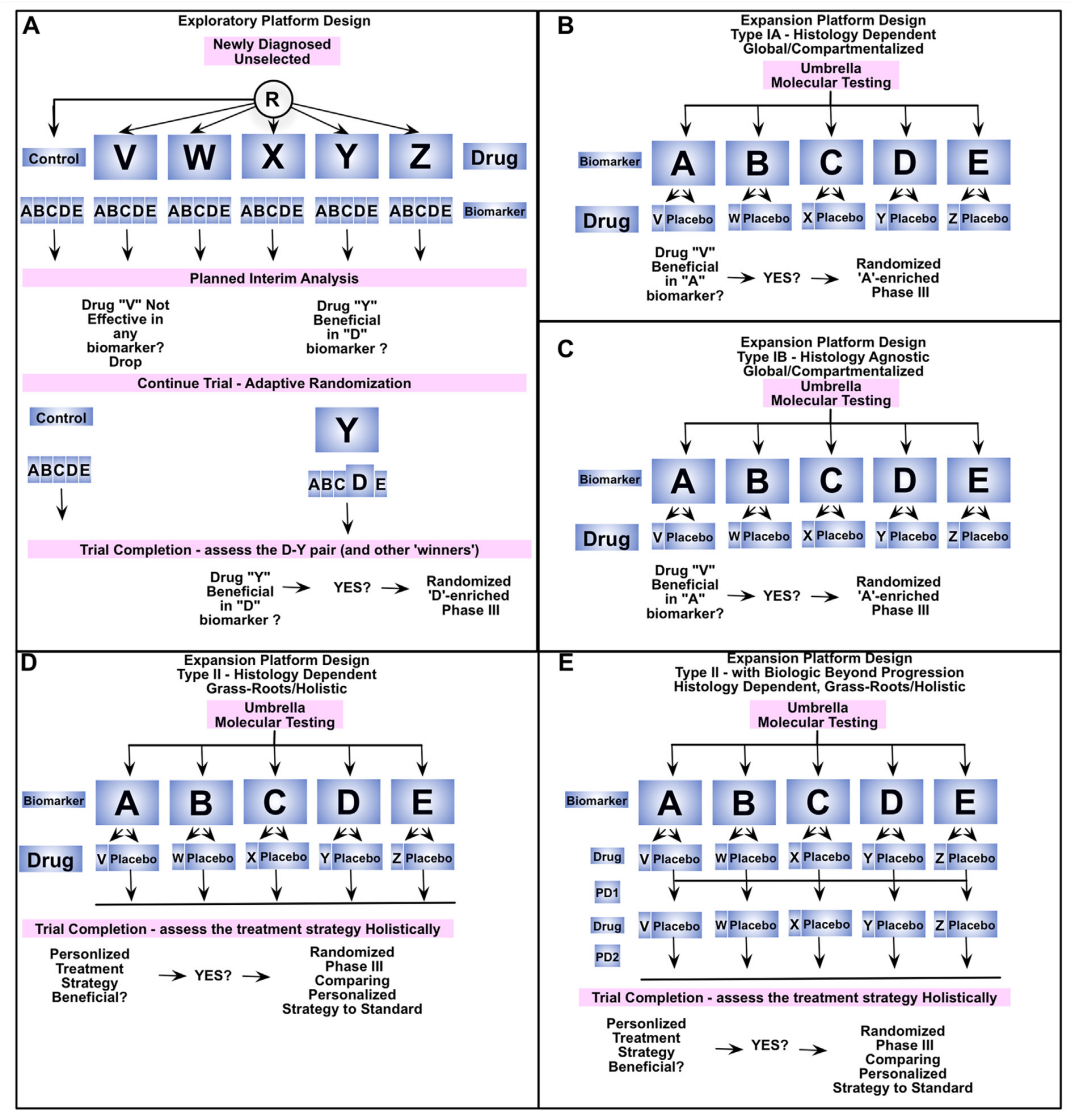
Next‐generation clinical trial designs. (A) Exploratory Platform Design (e.g. ‘BATTLE’, ‘I‐SPY’). (B) Expansion Platform Design Type IA: Histology Dependent, Global, and Compartmentalized. (e.g. ‘FOCUS‐4’) (C) Expansion Platform Design Type IB: Histology Agnostic, Global, and Compartmentalized (e.g. ‘NCI‐MATCH’, ‘Signature’). (D) Expansion Platform Design Type IIA: Histology Dependent, Grass‐Roots, Holistic (e.g. PANGEA). (E) Expansion Platform Design Type IIB with Biologic Beyond Progression: Histology Dependent, Grass‐Roots, Holistic (e.g. PANGEA‐BBP). After first progression (PD1) patients undergo repeat biopsy of a progressing lesion and undergo repeat molecular testing and treatment assignment, which may allow cross‐over to a more appropriate biological group as directed by the prioritization algorithm (Figure 7). Patients on placebo remain on placebo at each progression point.

In particular instances where there is uncertainty as to the optimal molecular subset(s) to apply a given therapeutic agent, the ‘Exploratory Platform’ design has emerged. Notable examples include the ‘BATTLE’ (Kim et al., 2011) and ‘I‐SPY’ (Barker et al., 2009) trials (Figure 5A). Using a Bayesian adaptive statistical approach, (Wang et al., 2012) drug arms (or ‘bins’) are pre‐specified, and patients are randomized to each arm evenly (ie. not ‘personalized’ initially). Molecular testing is performed on the tumor biopsy prior to randomization and the pre‐specified biomarker subsets are stratified evenly between the drug cohorts (an integrated biomarker design, initially). This design is essentially an umbrella biomarker stratified design (Figure 4D) (if a control arm is included – I‐SPY 2 does, BATTLE didn't), exploring several drug cohorts and a number of molecular biomarker subgroups simultaneously. Over time, if a significant efficacy signal is identified in a certain biomarker‐drug pair at a planned interim analysis, then adaptive randomization of that now integral biomarker (e.g. molecular profile D) to that treatment arm (e.g. treatment Y) ensues (Figure 5A). (Blumenschein et al., 2013; Tsao et al., 2013; Po, 2014; Ne, 2014) Alternatively, drugs that do not demonstrate benefit in any of the molecular profiles are dropped. If molecular profile D and treatment Y continue to demonstrate the initially observed signal through the later trial stages, this newly recognized pairing of ‘profile D → treatment Y’ can be confirmed statistically in a traditional ‘population enriched’ phase IIb trial or may proceed directly to a phase III confirmatory trial (Figure 4B), with all the advantages/disadvantages that this design entails, as discussed above with population enriched trials (Table 4, Figure 8).

#### 5.1.2 Expansion platform Type IA: Global, Compartmentalized & Histology Dependent (e.g. ‘FOCUS‐4’)

The ‘FOCUS‐4’ trial, (Kaplan et al., 2013) recently described for advanced colon cancer, is a first example of this clinical trial design, referred to herein as an ‘Expansion Platform Type IA’ trial; this is a global, compartmentalized, and Histology Dependent trial, ‘expanding’ from the population enrichment design evaluating one biomarker, (Figure 4B), into a platform consisting of multiple biomarker categories. This overarching ‘umbrella’ protocol design provides structure in which there can be global molecular characterization of patient tumors into specific integral biological cohorts. The ‘FOCUS‐4’ trial includes: Cohort A, *BRAF* mt; Cohort B, *PIK3CA* mt and/or PTEN loss; Cohort C, *RAS* mutation; Cohort D, All wild type, and also includes a molecularly ‘unclassified’ or ‘relegation’ Cohort E (Figure 5B). In contrast to the Exploratory Platform discussed above, the Type IA Expansion Platform identifies integral molecular subsets prior to trial initiation, (Dancey et al., 2010) and assigns therapy to only those within that biological subset. The example of the ‘FOCUS‐4’ trial applies this Type IA design for maintenance therapy after obtaining stable disease (or better) on first line therapy for stage IV colorectal cancer. It also includes a treatment prioritization scheme to address the issue of multiple biomarkers found within one individual (ie. Cohort A > B > C > D > E). The Expansion Platform Type IA design is differentiated from the Type IB design, below, which is histology‐agnostic. The Type IA is differentiated from Type II designs, below, in that each cohort in the Type IA is run as a separate (compartmentalized) phase IIa (or ideally randomized phase IIb) trial with distinct Principal Investigators, and isolated statistical endpoints for each subgroup. This Type IA Expansion design requires a global ‘top‐down’ organizational infrastructure, such as is possible as this large national study in the United Kingdom. This global approach requires the inclusion of multiple enrolling centers, to increase accrual rate to the rare molecular subsets in order to achieve statistical power *within each subgroup*. Cooperative Groups and NCI/CTEP in the USA could also design trials in this fashion as well, such as the planned ‘MASTER PROTOCOL’ for squamous cell lung cancer, (Abrams et al., 2014) as could pharmaceutical companies (alone or in collaboration) able to embody multiple biological cohorts within their pharmaceutical portfolio.

#### 5.1.3 Expansion platform Type IB: Global, Compartmentalized & Histology Independent (e.g. ‘NCI‐MATCH’ and the Novartis ‘Signature’ trials)

The Expansion Platform Type IB design is also an umbrella global design with the main difference from the Type IA being that it is histology‐agnostic (Figure 5C). Trials and concepts to date following this design include the ‘NCI‐MATCH’ ‘basket’ protocol (currently planning to screen ∼3000 patients and treat 1000), (Abrams et al., 2014) which will include multiple histology‐agnostic phase IIa (or ideally phase IIb) arms each consisting of an identified genomic aberration(s) and ‘matched’ targeted therapy. A second trial example is the ‘Signature’ ‘basket’ trial by Novartis currently with five molecular ‘bins’, each registered as an individual phase IIa (non‐randomized) NCI trial. To date, neither of these two examples have specified a treatment prioritization algorithm, if a patient were to be eligible for more than one molecular bin, however, NCI‐MATCH will putatively submit profiling results to the ‘automated MATCHBOX rules engine’ where aberrations will be matched to treatments based on a currently undisclosed prioritization scheme. A question remaining is whether follow up phase II/III randomized trials would be required for FDA approval of ‘successful’ single arm phase IIa trials that have primary endpoints of response rate or disease control rate. If so, it is obvious that the numbers of patients required from start (phase IIa) to the completion of the biomarker selective population‐enriched phase III trial are vast (Figure 8). However, the recent example of crizotinib gaining FDA approval for *ALK‐*translocated NSCLC without a traditional randomized phase III trial may have emboldened this single arm phase IIa approach.

#### 5.1.4 Expansion platform Type IIA: Grass‐Roots, Holistic & Histology Dependent (e.g. ‘PANGEA’ trials)

The Expansion Platform Type IIA design, on the other hand, is a trial design that is not global, nor compartmentalized, and can be considered a ‘Grass‐Roots’ or investigator initiated approach. In fact, it can be performed in single institutions as a pilot trial (phase IIa non‐randomized) or within smaller collaborative groups as randomized phase IIb trials (Figure 5D). The ‘PANGEA’ concept (Personalized Antibodies for Gastro‐Esophageal Adenocarcinoma) is the first example of the Type II holistic design. (Catenacci et al., 2014a) As in the Type IA Expansion Platform, the Type IIA ‘PANGEA’ design identifies various integral molecular subsets within GEC that are tiered by level of priority and degree of anticipated benefit from targeted inhibition (Figure 7) prior to trial initiation. Therapy is assigned specifically only to those within that biological subset. This treatment assignment is based on current understanding of ‘driver’ biology of that tumor type at the time of trial initiation, matching available targeted therapies thought to best suit each molecular subset. It also relies on a pre‐specified treatment prioritization algorithm to address multiple ‘drivers’ and inter‐patient heterogeneity (Figure 7, Table 3), (which could be considered arbitrary, particularly if anticipated hazard ratios are similar between different potential biomarker‐drug pairings). Should multiple aberrations be present, priority in ‘PANGEA’ will be given to higher allele frequency (for mutations) or higher gene copy/expression, (Gomez‐Martin et al., 2013) for example. However, the Type IIA design is executed as one uniform (holistic) umbrella trial, with one primary statistical endpoint testing the hypothesis that personalized therapy is better than the current standard therapy for that tumor type – it is ultimately testing the treatment strategy comprised of numerous companion diagnostics and their respective matched targeted therapies. All patients screened are eligible irrespective of their molecular profiling result, due to relegation tier(s) within the treatment algorithm (Figure 7). The design can be applied at any line of therapy to evaluate a ‘personalized strategy’ compared to the standard treatment for that scenario. It can be performed as a phase IIa pilot compared to historical outcomes, as a randomized placebo‐controlled phase IIb, or conceivably even as a large registration phase III trial (for the pre‐specified treatment strategy), if warranted, based on promising phase IIb trial results (Table 4, Figure 8).

#### 5.1.5 Expansion platform Type IIB: with Biologic Beyond Progression (e.g. ‘PANGEA‐BBP’)

**Figure 6:**
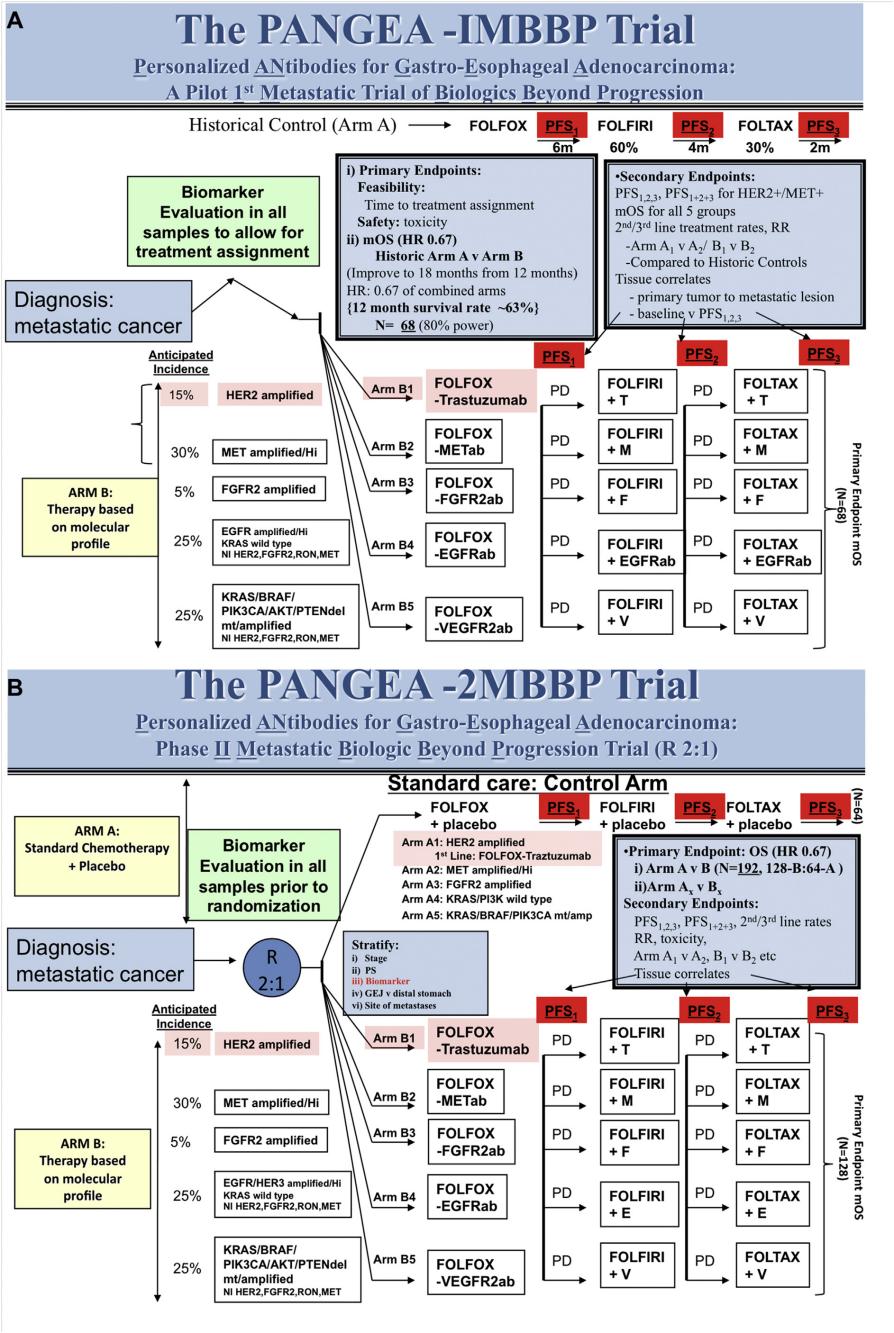
The ‘Expansion Platform Design Type II with BBP – PANGEA’. (A) Schema of the ongoing pilot ‘phase IIa’ trial called ‘PANGEA‐IMBBP’. (B) A planned future randomized placebo‐controlled phase IIb trial ‘PANGEA‐IIMBBP’ should the pilot trial meet endpoints. Molecular categorization is a stratification factor to ensure equal distribution between Arms A and B. HER2+ patients would receive trastuzumab in the first line, per clinical standards, then proceed with placebo for second/third line therapy.

None of the biomarker directed trials discussed thus far systematically address (with action) intra‐patient tumor heterogeneity though time. All are focused on one line of therapy, and none control post‐progression treatment (in either the placebo or treatment arms) which may result in confounding when evaluating an overall survival outcome, as has been experienced in numerous trials, including the current debate surrounding discordance between the ‘FIRE‐3’ and ‘CALGB‐80405’ colorectal trials. (Catenacci et al., 2011c; ESMO, 2014) The Expansion Platform Type IIB design *with* Biologic Beyond Progression (BBP) builds on the Expansion Platform IIA, by incorporating a sequential biopsy after failure of therapy (or through multiple lines of therapy), along with a reassigned targeted therapy as appropriate (Figure 5E). (Catenacci et al., 2014a) This is an attempt to address tumor evolution and resistance over time. The first iteration of the ‘PANGEA’ trial (IMBBP, NCT02213289) is an example of this Type IIB Expansion Platform Design, seeking to address the hurdles posed by *both* inter‐ and intra‐patient tumor molecular heterogeneity and attempting to realize the full potential of targeted therapeutics (Figure 6A).

## 6 ‘PANGEA‐BBP’ and the ‘Biomarker & Treatment Assignment Algorithm’

**Figure 7:**
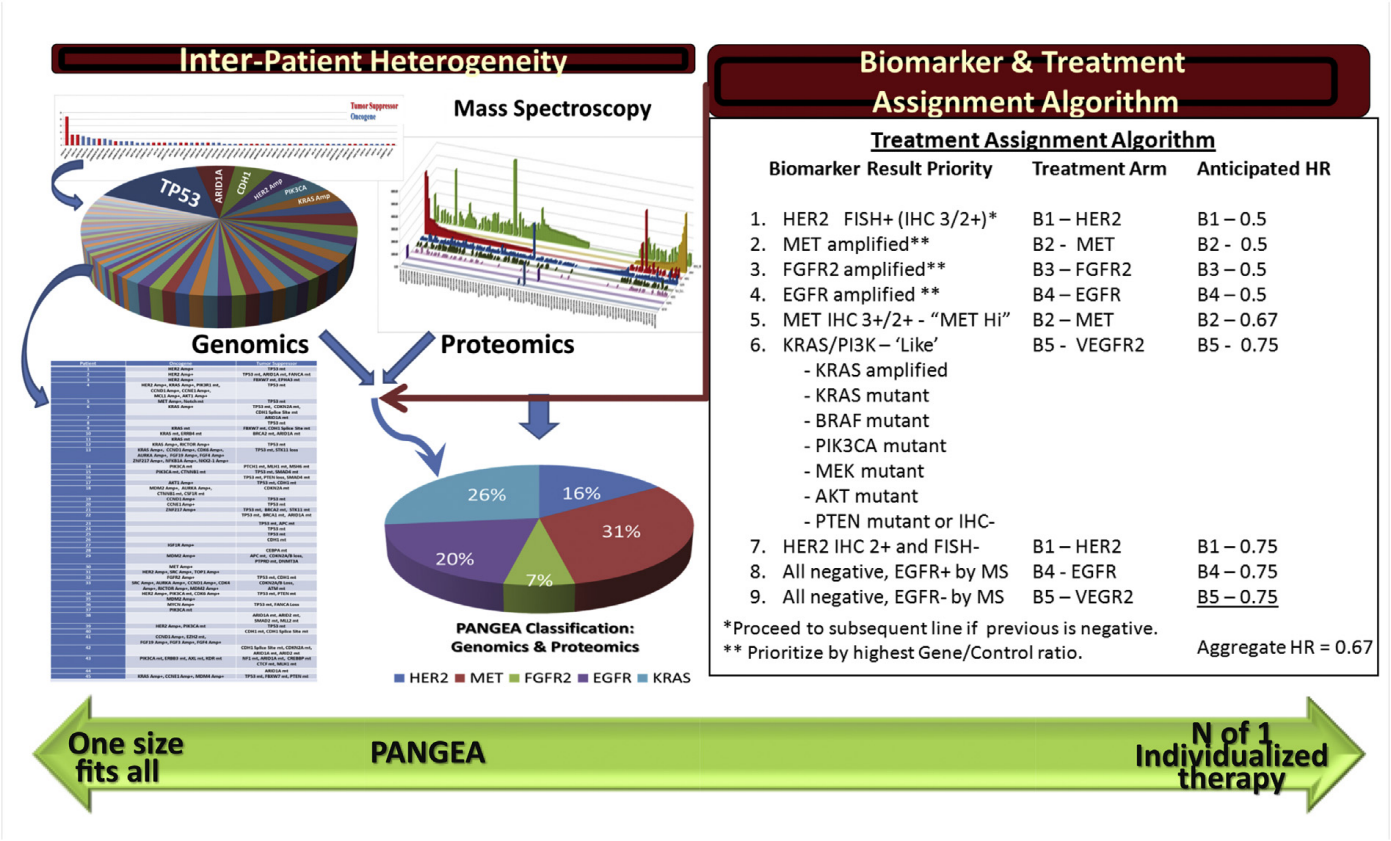
The biomarker and treatment assignment algorithm is premised on optimizing the inhibition of ‘driver‐biology’. This 9‐point algorithm serves to prioritize treatment assignment should multiple aberrations (genomic and proteomic) be observed in an individual sample. Should multiple aberrations be present, priority could be given to higher allele frequency (for mutations) or higher gene copy/expression. The algorithm acts as a filter to create 5 distinct biomarker categories (with 9 tiers) that will receive 5 specific and most‐appropriately matched targeted therapies. Approximate hazard ratios (HR) anticipated for each categorized tier, as well as the aggregate HR (the primary endpoint of PANGEA), are indicated. This first iteration of the ‘PANGEA’ strategy is a compromise within the spectrum between the two extremes of ‘one‐size‐fits‐all’ and completely individualized therapy or ‘N‐of‐1’ (bottom panel). Rather than being a ‘tailored suit’, PANGEA can be considered fitting to ‘X‐large, large, medium, small and X‐small’. Future iterations could include more biomarker categories and treatment arms, consequently moving closer towards the ‘N‐of‐1’ limit.

The ‘PANGEA‐IMBBP’ trial (Figure 6A), is a pilot (phase IIa) trial for patients with GEC evaluating a personalized treatment algorithm that is compared to historical control survivals of approximately 12 months (NCT02213289). (Catenacci et al., 2014a) Ultimately, should the initial feasibility, toxicity, and early efficacy endpoints be met on this pilot, the trial will proceed to a placebo‐controlled randomized phase IIb (‘PANGEA‐IIMBBP’) to evaluate the primary endpoint of overall survival (Figure 6B). In this particular iteration of the Type IIB design, the targeted therapies chosen for ‘PANGEA’ are monoclonal antibodies, due to the benefit of antibody specificity, ADCC, and ease of combination with cytotoxics for this tumor type, facilitating use in first line setting (Table 1). Given the recognized inter‐patient molecular heterogeneity (Figure 3, Table 3), choosing five or six biologic cohorts is largely a compromise between the feasibility of acquiring various targeted drugs for each group and the putative number of potential biological cohorts. Rather than being a ‘tailored suit’, PANGEA can be considered fitting to ‘extra‐large, large, medium, small and extra‐small’. The ‘PANGEA’ ‘Biomarker & Treatment Assignment Algorithm’ prioritization scheme is the key variable being tested in this trial (Figure 7, Table 5). The BBP component of this Expansion Platform Type IIB design will mandate biopsies at each progression time‐point to evaluate tumor molecular evolution (heterogeneity through time). In addition to altering the standard backbone cytotoxic therapy at each time of progression as is routinely done clinically, assessment will be made on the ‘real‐time’ biopsy to either continue the previously assigned biologic (if the tumor has not evolved to a different biologic subgroup) or to change to a different biologic (if the tumor has evolved to a different biologic subgroup). Evidence that maintenance of ‘biologic inhibitory pressure’ on an oncogenic ‘driver’ is effective despite first (or second) progression has been demonstrated both pre‐clinically and clinically in various settings. (Cepero et al., 2010; Grothey et al., 2008; von Minckwitz et al., 2009; Verma et al., 2012) On the other hand, should there be clear ‘migration’ of ‘driver’ status within the prioritization algorithm to a new biomarker classification at any progression time‐point, the patient will be treated with the new appropriate biologic therapy matched to the new molecular subgroup. In this pilot ‘phase IIa’ study, multiple phase‐Ib‐like safety lead‐ins for each biologic/cytotoxic pairing will be done if full phase I data is not yet established, rather than separate large phase Ia trials; it is anticipated that the addition of antibodies to various cytotoxic regimens will not substantially alter safety profiles. At each disease progression time‐point, the ‘PANGEA‐IMBBP’ iteration in Figure 6A allows for treatment cross‐over based on the ‘Biomarker & Treatment Assignment Algorithm’ – but a trial could be designed without such allowance, with the previous biologic continued despite evolution while altering the chemotherapy backbone, or by continuing the original biologic, but adding a second (Table 5). Also, the ‘PANGEA‐IMBBP’ iteration maintains a constant ‘Biomarker & Treatment Assignment Algorithm’ at each progression time‐point, but could alternatively test the application of a ‘dynamic algorithm’, in attempt to ensure a different biologic therapy at each progression point. More complex designs with combinations of biologics could also be envisioned (Table 5), as it is possible that ‘personalized therapeutic cocktails’ may ultimately be required to optimally inhibit molecularly heterogeneous tumors.

**Table Table 5:** Characteristics, options, and variables within the design of next‐generation clinical trials.a

Variable/Characteristic	Exploratory platform design e.g. ‘BATTLE‐1’	Expansion platform design type IA e.g. ‘FOCUS’	Expansion platform design type IB e.g. ‘Signature’	Expansion platform design type II e.g. ‘PANGEA‐IM’	Expansion platform design type II with BBP e.g. ‘PANGEA‐IMBBP’
Reflecting this ‘Classic’ biomarker design:	Biomarker stratified	Population enriched	Population enriched	Population enriched	Population enriched
		Histology dependent	Histology agnostic	Histology dependent	Histology dependent
Biomarker enriched:	NA	Compartmentalized	Compartmentalized	Holistic	Holistic
Compartmentalized vs holistic		Testing each group with individual endpoints and stats	Testing each group with individual endpoints and stats	Testing the personalized treatment strategy	Testing the personalized treatment strategy
No. Biomarker groups	4	5	5 (more planned)	5	5
Biomarker groups	1 EGFR mt 2 KRAS/BRAF mt 3 VEGF/VEGFR2 4 RXR/CCND1	1 BRAF mt 2 PIK3CA mt/PTEN‐ 3 RAS mt 4 All Wild type 5 None of above	1 PIK3CA mt/PTEN‐ 2 FGFR/PDFR/VEGF/FLT3/CSFR1/TRKA/RET 3 RAS/MEK/NF1 4 BRAF 5 SMO/PTCH1	1 HER2 2 MET 3 EGFR 4 FGFR2 5 RAS/PI3K ‘like’	1 HER2 2 MET 3 EGFR 4 FGFR2 5 RAS/PI3K ‘like’
Targeted agents	1 erlotinib 2 vandetanib 3 erlotinib + bexarotere 4 sorafenib	1 TBD 2 TBD 3 TBD 4 TBD 5 capecitabine	1 buliparib 2 dovitinib 3 Mek162 4 LGX818 5 LDE225	1 anti‐HER2 Ab 2 anti‐HGF/MET Ab 3 anti‐EGFR Ab 4 anti‐FGFR2 Ab 5 anti‐VEGFR2 Ab	1 anti‐HER2 Ab 2 anti‐HGF/MET Ab 3 anti‐EGFR Ab 4 anti‐FGFR2 Ab 5 anti‐VEGFR2 Ab
Targeted agent properties	TKI	TBD	TKI	Monoclonal Ab	Monoclonal Ab
No. targeted agents per group	1 or 2	TBD	1	1	1
Combination with standard therapies	No	No	No	Yes	Yes
Line of therapy	≥2L	Maintenance after 1L	≥2L	1L	1L→2L→3L
Phase of proposed trial	Phase IIa	Phase IIb	Phase IIa	Phase IIa	Phase IIa
Estimated total patients required for screening for the trial actually proposed (see Figure 8)	341 (Battle‐1)	∼1375 (∼275/arm × 5)	350 (70/arm × 5)	68	68
	450 (Batte‐2)				
	800(I‐SPY 2)	(180 × 5 = ∼900if phase IIa)	(NCI‐MATCH ∼3000 patients screened for ∼1000 enrolled)		
Primary endpoint	DCR at 8 weeks	PFS	CBR at 8 weeks	PFS	OS
Randomization ratio for proposed trial (2:1 etc)	NA	TBD	NA	NA	NA
Potential for RCT Phase IIb and placebo with this design	Yes if control group included (e.g. I‐SPY 2 standard therapy arm)	Yes	Yesb	Yes	Yes
Future phase IIb required?	Yes	No	Yes	Yes	Yes
		(optional biomarker‐stratified)c		Phase IIb randomized 2:1 for future trial	Phase IIb randomized 2:1 for future trial
Future phase III confirmatory trial?	Yes	Yesc	Yesb	Yesd	Yesd
Future phase III trial design type	Classic biomarker enriched(Histology dependent)	Classic biomarker enriched(Histology dependent)	Classic biomarker enriched(Histology agnostic)	Expansion platform Type IIA(Histology dependent)	Expansion platform Type IIB‐BBP(Histology dependent)
Total patients to be screened for eligibility	Manye	∼2500 (∼550/arm × 5)f	Manyg	∼600d	∼600d
All‐comers included	Yes	Yesk	No	Yes	Yes
Coordination	Global	Global	Global	Grass‐Roots	Grass‐Roots
Treatment assignment prioritization algorithmh	Norandom assignment, then adaptive randomization	Yesh	No	Yesh	Yesh
Addresses resistance within the same trial	No	No	No	No	Yes (i.e. BBP)
If yes, BBP treatment priority algorithm	NA	NA	NA	NA	Yes
If yes, treatment algorithm same as prior line (static)i or altered (fluid)j	NA	NA	NA	NA	Static algorithm
Trial design subject to confounding of OS endpoint due to post‐trial treatment	NA	Yes	Yes	Yes	No
Ability to drop/add biomarker groups and/or paired drugs on a rolling basis	Yes	Yes	Yes	NoRefine future trials based on previous completed trials	NoRefine future trials based on previous completed trials

BBP, biologic therapy beyond progression; NSCLC, non‐small cell lung cancer; GEC, gastro‐esophageal adenocarcinoma; mt, mutant; PTEN‐, PTEN loss; TBD, to be decided; Ab, antibody; TKI, tyrosine kinase inhibitor; 2L, second line; 1L, first line; 1L→2L→3L, including first, second and third line therapy; DCR, disease control rate; PFS, progression‐free survival; CBR, ‘clinical benefit rate’ (=∼ DCR); OS, overall survival; NA, not applicable.

a Numerous variables within each trial design can distinguish different trials within the same design (e.g. BATTLE‐1 vs BATTLE‐2 vs I‐SPY 2 within the Exploratory Platform Design), while specific variables may not be applicable to certain design types. Representative trials within each trial design type are exemplified with their actual characteristics.

b Difficulty in randomizing patients with various tumor types complicates randomized histology agnostic trials, for both phase IIb and Phase III designs. Stratification by tumor type may be plausible, but if combining with standard therapies (e.g. if first line) greatly complicates this design since standard therapies are diverse across tumor types.

c A biomarker stratified design (phase IIb) could follow the phase IIb biomarker enriched design (if there is question as to benefit of the investigational drug in the biomarker‐negative patients), or it could proceed directly to a confirmatory phase III population enriched design.

d A phase III trial could be designed holistically testing ‘personalized treatment’ versus control, pooling the subgroups together towards the primary endpoint, with the advantage of requiring significantly fewer patients. The caveat is that all biomarker subgroups along with matched targeted agents chosen must contribute to the overall benefit observed (ie. the HR for each subgroup, although not required to be equal, should all be < 0.8, and the aggregate HR must meet the primary overall endpoint – see Figure 7). The power to detect benefit of each subgroup will be limited, unless the benefit observed is large.

e Depending on the frequency of the biomarker subset within the population studied (Histology Dependent), the ability to identify the benefit in that subgroup, if small, in the exploratory platform design may not have adequate power, unless initial trial sizes are substantially larger. Moreover, once the second adaptive randomization phase establishes benefit (which requires more patients), the confirmatory phase III trial would still require very inflated numbers of patients screened to identify the infrequent biomarker + patients.

f Screening 2500 patients (after the initial 1375 patients in the phase IIB) may be plausible with global coordination (eg Research UK and Medical Research Council Clinical Trials Unit) for a high incident tumor such as colorectal cancer. However, many tumor types do not have this ‘luxury’ of high incidence and would have difficulty with such high numbers required for screening/accrual.

g Depending on the frequency of the biomarker subset within the population studied (histology Independent), the ability to identify benefit in a phase IIb trial would require high patient numbers to identify infrequent biomarkers (despite searching across tumor types), and the ensuing confirmatory phase III would also need many patients. Also see point b above regarding histology agnostic designs.

h The treatment assignment algorithm is an effort to address inter‐patient heterogeneity and multiple concurrent aberrations within the tumor sample. Despite best efforts to incorporate current biologic understanding and rationale, ultimately this algorithm is arbitrary.

i Intra‐patient tumor heterogeneity over time (ie treatment resistance) can be assessed by repeat biopsy (or surrogate via serum/urine assays) of a progressing lesion, re‐evaluating biomarker status, and re‐assignment by the treatment algorithm. Allowing cross‐over to the new biomarker/drug group as appropriate may enhance the personalized strategy.

j The treatment algorithm can be held constant at each progression time‐point in the Type BBP design, (e.g. if there is still HER2 amplification, but a newly acquired MET amplification observed at the time of progression, continued anti‐HER2 therapy would be indicated because HER2 is first in the priority tree), or the algorithm could be fluid (e.g. if in the same scenario, if there has been progression on anti‐HER2 therapy, a fluid algorithm could exclude eligibility from the prior biomarker group, and proceed to the next groups, or a fluid algorithm could allow for continued anti‐HER2 but also addition of other targeted therapies directed at the newly acquired molecular aberration, in this case an anti‐MET agent.).

k FOCUS‐4 includes a relegation cohort (E) that is negative for inclusion in cohorts A‐D. However, other Type IA designs do not necessarily include such a relegation category (e.g. NCI‐MATCH), and therefore not all screened patients would be eligible.

The challenges of a single institution or Principle Investigator contracting with several pharmaceutical companies for participation in any of the Expansion Platform Designs cannot be underestimated (it is not an understatement that ‘PANGEA’ was attempting to ‘move continents’ going on five years from conception in 2009 to opening 2014!), but this has become increasingly more feasible. Similarly, issues surrounding validation of next‐generation companion diagnostics prior to trial initiation are also prohibitive, but achievable, as discussed below. (Khoury and Catenacci, 2014) Statistical considerations of Type II Expansion Trials, for phase IIa, phase IIb and phase III will be reported separately (other than the major clinical endpoints shown in 6, 8). The detailed statistical methods for ‘PANGEA’ include pre‐specified statistics in the phase IIb and/or phase III settings to i) identify prognostic implications of each molecular group as determined by placebo‐controlled stratification of each biomarker subgroup; and ii) identification of significant interactions on clinical outcome between and within the molecular subsets and matched therapies, to assist in determining future trial iterations and optimal treatment approaches.

## 7 Next‐generation clinical trials: a comparison

**Figure 8:**
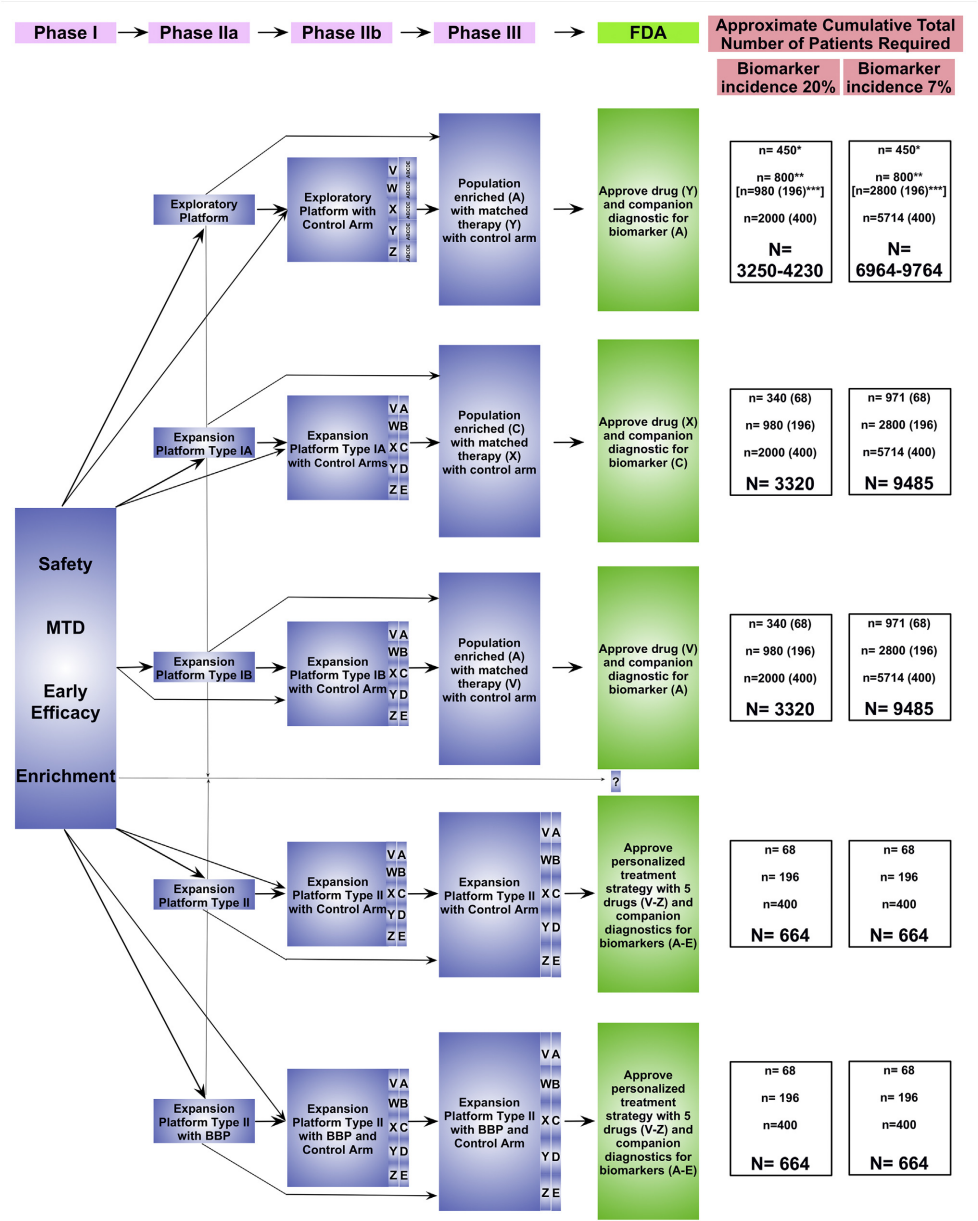
Applications of next‐generation clinical trial designs, and total patients required, towards approval of ‘personalized’ treatment strategies that encompass both the drugs and companion diagnostics. Total numbers of patients required from phase II to phase III and FDA approval are approximated in the final right column, using a biomarker incidence of 20% and 7% as examples. For comparison purpose, the numbers reflect a median overall survival as the primary endpoint with target HR 0.67, two‐sided alpha 0.05, 80% power, randomization ratio 2:1, 12 month accrual and 24 month follow up. Total numbers for each trial design include estimated numbers for serial phase IIa, phase IIb, and then phase III trials in tandem. For the exploratory platform design, given the adaptive Bayesian statistics, a direct comparison is not possible. * The target total number for the ongoing BATTLE‐2 trial. **The target total number for the ongoing ISPY‐2 trial. ***Estimated numbers for a follow up randomized phase IIb trial for an identified biomarker/drug combination from either the phase IIa or Phase IIb Exploratory Platform design, with statistical endpoints as set above, performed prior to a full phase III. Numbers in parentheses indicate the target biomarker population subset that would be required to be identified from the entire patient population.

Advantages and disadvantages of the discussed ‘Next‐Generation’ trials are detailed in Table 5. Different settings will call for application of different trial designs (Figure 8). Different diseases may likely need to be approached differently, and diverse health care systems may dictate feasibility of one approach versus another. Retrospective‐prospective trials remain reasonable for already‐completed trials to explore translational concepts. Classic ‘histology‐dependent population‐enriched’ trials may be useful for biomarkers that are relatively prevalent, but will not be ideally suitable for low frequency molecular subsets. ‘Histology‐independent population‐enriched’ trials continue to be used for early signal detection in rare molecular subsets in ‘phase Ic expansion’ trials, which are essentially multiple parallel tumor‐specific phase IIa trials (e.g. anti‐PDL1 for ∼40 patients of each tumor‐type of interest within a ‘phase I expansion’) (Figure 8). A path to FDA approval for this strategy is not clear, yet if a substantial clinical benefit is observed compared to accurate historical controls when no alternative therapy exists, even in relatively small patient numbers, then approval may be possible (e.g. crizotinib and *ALK* translocation). Traditional ‘biomarker stratified’ designs performed ‘à la carte’ will likely become obsolete with the recognition of smaller and smaller molecular subsets, requiring too many negative screens and inefficient use of precious tissue samples and screening time. (Warth et al., 2012) Molecular profiling from the peripheral blood may ameliorate this problem, as discussed below, but this is currently not validated nor readily available for clinical application.

‘Exploratory Platform Designs’ require large numbers of patients, along with significant coordination, pharmaceutical participation, and funding, yet they may be useful to identify molecular profiles that predict benefit to specific drugs in cases where strong preclinical/clinical evidence establishing such associations is lacking. In general, for Exploratory Platform designs, performed in phase IIa settings, these will require spin‐off of newly discovered biomarker‐drug pairings through prospective placebo‐controlled randomized phase IIb and/or III trials (requiring even more patients) for confirmation and ultimate approval (Figure 8).

Type IA and IB Expansion Platform Designs will require extensive top‐down coordination, pharmaceutical participation, and companion diagnostic validation, all of which can make this difficult to materialize. However, if in place, such as the first five modules of the ‘Signature Trial’ (now 8 trials just prior to manuscript publication) this umbrella screening protocol will funnel patients into similar biologic groups having distinct ‘stand‐alone’ trials assessing relevant biomarker(s)‐drug(s) pairing questions. However, the numbers of patients required to proceed from Phase IIa→IIb→III confirmation and ultimately regulatory approval are drastically high (Figure 8). For the histology‐agnostic Type IB trial, the assumption that a molecular aberration behaves identically across histologies may not be true, exemplified by *BRAF* mutation and differences in clinical benefit in melanoma and colorectal cancer with inhibitory BRAF monotherapy. Genomic events in the context of tumor histology may prove to be important going forward in more examples. The Type IB Phase IIa trials done with exploratory intent may help to elucidate this question. Backbone chemotherapies differ across tumor types, posing challenges for the Type IB trial design when attempting to evaluate molecularly targeted therapies in combination with cytotoxics in earlier lines of therapy.

**Figure 9:**
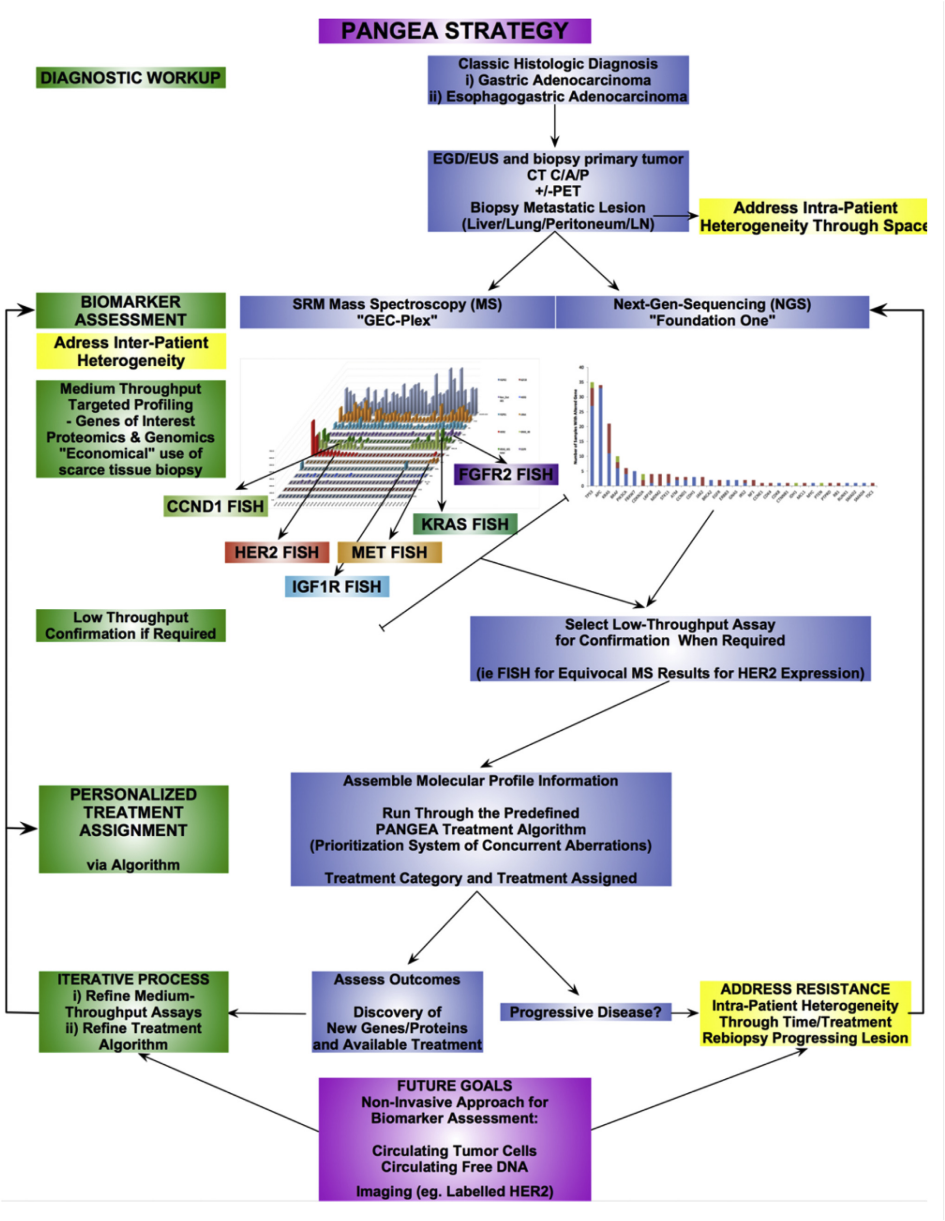
The ‘PANGEA’ strategy addressing inter–and intra–patient tumor molecular heterogeneity. The expansion platform type II design with biologics beyond progression is testing the ‘PANGEA personalized treatment strategy’. Obtaining baseline biopsies of metastatic disease and serially biopsies at each progression time‐point within the trial with repeat molecular testing and treatment assignment to match targeted therapies in real‐time may improve clinical outcomes, compared to a historical (phase IIa) or placebo (phase IIb) controlled standard therapy. Upon completion of each trial, an iterative process will allow to refine the treatment strategy (biomarker assays, molecular categories, treatment algorithms, and therapeutic agents) using knowledge gained from each previous trial and new technology and drugs developed in the interim.

The Type II Expansion Platform Design (with or without BBP) is ideal for small single institution Phase IIa pilot studies (*n* = ∼70 for HR 0.66) and even Phase IIb studies (*n* = 100–200, depending on the HR, power, type II error, and randomization ratio desired) that assess one strategic approach of several relevant biomarker‐drug pairings (Figure 8). It can even conceivably test the ‘personalized treatment strategy’ as a stand‐alone phase III trial with intent for regulatory approval for all components (companion diagnostics, drugs, and treatment algorithm) within the strategy, or could be spun off to have individual phase III trials for each molecular subset should a signal be identified in one of the biomarker‐drug pairings, with the caveat of substantially increasing the numbers of patients required to reach individual subgroup statistical endpoints (Figure 8). This Type II trial design is dependent on a host of factors including accurate biomarker‐drug pairings, validated companion diagnostics, and most specifically a correct treatment algorithm. However, given the low numbers required for pilot trials, numerous iterations can be conducted across various centers altering any of a number of variables within the design of the trial (Table 5). This iterative process (Figure 9) along with consequent refining of biologic subgroups, better matched therapies (or use of other agents within a given drug class), and a whole host of other variables (Table 5) will allow for multiple centers, cooperative groups, and pharmaceutical companies to tackle these questions simultaneously. Smaller pharmaceutical companies can also participate within limited budgets, as relatively fewer patients would be assigned to their actual treatment arm within the trial, and the umbrella screening costs would be shared by all trial pharmaceutical participants. Ultimately, the phase IIa and IIb Type II Expansion trials that identify the most promising holistic approaches, could ideally ‘expand’ to a phase III Type II Expansion trial to test the treatment strategy as compared to the prevailing standard therapy. Should the new personalized treatment strategy meet pre‐specified survival endpoints with statistical significance compared to control, this would be grounds to consider it as a new standard of care (Figure 8). Future iterations could then compare, for instance, ten refined molecular subgroups compared to the newly established personalized approach with five subgroups, in the example of ‘PANGEA’. Smaller increments of progress (starting with 5 subgroups, then moving to 10 etc), as opposed to attempting 10 or more right away, will limit the problems concerning uncertainty of benefit across each and every one of the included subgroups (Figure 7).

## 8 Next‐generation companion diagnostics

Attention to the regulation of companion diagnostics increased dramatically after the investigations regarding the ‘Duke scandal’ came to light. (Kurzrock et al., 2014) A recent opinion letter detailed the difficulties in achieving optimal balance between imposing FDA regulatory oversight and facilitating advances in the development of innovative biomarker testing and trial designs, (Kurzrock et al., 2014) and many have called for a complete overhaul of the regulatory system for diagnostic tests. (Hirsch et al., 2014) Multi‐plex analysis of biomarkers will be required to overcome the hurdle of inter‐patient molecular heterogeneity and limited tissue, as discussed above. (Stricker et al., 2011; Khoury and Catenacci, 2014; Collins and Hamburg, 2013) Multivariate analyses associating combined genomic and proteomic profiles with clinical outcomes will likely assist to optimally determine best uses of each targeted therapy. (Catenacci et al., 2014b; Parkinson et al., 2012; Mendelsohn, 2013; Garraway et al., 2013; Hembrough et al., 2012) Clear paths to regulatory approval for pre‐specified companion diagnostics and drugs within the Expansion Platform Type II trial design, such as a future ‘PANGEA‐IIIMBBP’, is paramount, as this will facilitate pharmaceutical collaboration in such trials (Figure 8). (Khoury and Catenacci, 2014) The approval of next‐generation DNA sequencing for marketing authorization recently by the FDA is a first step. (Collins and Hamburg, 2013) We recently proposed a certification mechanism for laboratories offering next‐generation companion diagnostics services coined “Certified Advanced Companion Diagnostics Facilities – CACDF” to meet certain benchmarks, such as performance against a standardized sample set, in order to be granted certification by the College of American Pathologists (CAP) under commission from the FDA. (Khoury and Catenacci, 2014) However, identifying genomic variants that can be validated *before* being integrated into decision making for purposes of modulating drug response are critical. (Khoury and Catenacci, 2014; Collins and Hamburg, 2013; Catenacci et al., 2014c) Future goals of next‐generation companion diagnostics using non‐invasive ‘liquid biopsies’ to assess for circulating tumor cells or free serum/urine DNA are already being assessed as potential surrogates to serial tumor biopsy (Figure 9). (Khoo et al., 2014; Diaz and Bardelli, 2014; Leary et al., 2012; Speicher and Pantel, 2014; Schwarzenbach et al., 2014) It may or may not be that the same information can be acquired from the peripheral blood and/or urine as from the tumor itself, yet there is great potential for one or both of these assays, and this needs to be tested with scrutiny. (Pantel and Alix‐Panabieres, 2013; Shimada, 2014; Alix‐Panabieres and Pantel, 2014) Ultimately, the least‐invasive strategy that best predicts therapeutic benefit of given targeted therapies at the lowest cost to the health care system will prevail.

## 9 Next‐generation FDA regulation: companion diagnostics and drug approvals

An initial concern of novel and complex trial designs, particularly the Type II Expansion Platform, is the exceedingly high number of variables embedded within the personalized strategy (Table 5). However, consider a hypothetically completed phase III ‘PANGEA‐IIIMBBP’, where the overall survival (OS) endpoint is met for the investigational arm of the schema in Figure 6B. Clearly, if the endpoint of OS is met in this hypothetical prospective, double‐blind, placebo‐controlled phase III trial, it can be strongly argued that the companion diagnostics, the biomarker subsets, their assigned treatments, and the pre‐stated treatment algorithm have, collectively, shown superiority over the standard control with statistical scrutiny (and had also done so prior in an earlier phase IIb). This is especially true if the pre‐specified subset statistical analyses verify that there are no identified ‘duds’. ‘Duds’ are defined as biomarker‐drug groups that actually do not provide benefit and do not contribute to the improved intention‐to‐treat survival of the holistic investigational arm (and may actually be detrimental). For example, what if the primary endpoint is met, yet the survival benefit is mostly due to only 2 of the 5 groups, while the other 3 biomarker‐drug groups are non‐contributing? As discussed earlier, pre‐defined statistics are intended to identify ineffective biomarker‐drug groups from progressing through the phase IIb into the confirmatory phase III schema, using an iterative process for planning future trial treatment strategies (Figure 9). Moreover, further scrutiny of each of the subsets within the phase III would also be planned to identify any ‘dud’ that was not identified at the phase IIb checkpoint. Certainly, the cross‐over nature of the BBP component of the Type IIB Expansion trial (if included in that design) does significantly confound the subgroup assessment for overall survival, while PFS1 would remain distinguishable prior to any cross‐over taking place.

**Figure 10:**
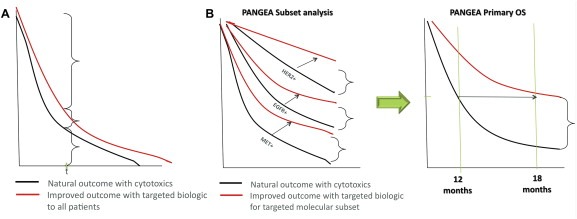
Comparison of one‐size‐fits‐all accepted design strategy and the ‘Expansion Platform Type II Holistic Design’. (A) In the classic clinical trial design, administering an investigational agent to all‐comers versus placebo will lead to approval, should statistical endpoints be met. Often, statistical endpoints are met with only marginal clinical improvement in overall survival (∼1–2 months). Approval of agents in this scenario leads to large numbers of patients treated with the new agent that do not derive any benefit (top and bottom bracket at any time‐point (t) along the x‐axis). Often targeted agents applied using this trial design fail since only a small subset derive benefit which is not recognized due to dilutional effects of the other biomarker‐negative patients, along with too few numbers within the subset analysis for adequate statistical power. (B) The Expansion Platform Design Type II (with/without biologics beyond progression) uses targeted agents for targeted populations (middle panel), in attempt to improve (red line) over the natural outcome observed for each specific molecular group treated without the targeted agent (black line). Three of the 5 subgroups of PANGEA are shown here as theoretical outcomes that are hypothesized. Due to the large number of patients that would be required should each of the molecular groups within PANGEA be run as an individual compartmentalized stand‐alone trial (ie an Expansion Platform Design Type IA or B), the advantage of the type II design is that all patients screened are placed in a group that is most appropriate for them within the one trial, reducing total patients required. Results are pooled (right panel) for the primary endpoint of ‘personalized treatment strategy’ versus standard control to limit exposure of any agent to any patient not expected to derive benefit, while maximizing exposure to those that will (bracket). Since the total effect size is hypothesized to be large, particularly in the higher tiers of the algorithm (see Figure 7), fewer total patients are required for statistical endpoints.

Regardless, many standard treatments to date are in place based on ‘one‐size‐fits‐all’ approaches of adding an investigational agent to standard therapy, where the benefit of adding the investigational agent was ‘statistically significant’, yet there were clearly more patients that derived *no* benefit at all (Figure 10A); yet it is considered ‘sufficient’ to set new treatment standards for *all* patients. For the Type II Expansion Platform Design, the personalized approach is hypothesized to derive higher absolute benefit collectively, since it is an aggregate of several targeted therapies used in targeted subgroups that are predicted to respond, prioritized in a manner to optimize clinical outcome (Figure 7). This may not be statistically evident when evaluating each molecular arm individually, due to lack of power (Figure 10B, left panel), but would certainly be apparent when evaluating the holistic approach in terms of aggregate benefit as the primary endpoint, the ‘raison d'être’ of the type II Expansion Platform Design (Figure 10B, right panel).

## 10 Conclusions

Tumor molecular heterogeneity is a hurdle to successfully realizing the full potential of molecularly targeted therapeutics. To date, ‘classic’ biomarker‐driven trial designs have attempted to address various aspects of molecular heterogeneity, but with recognized limitations. Next‐generation clinical trial designs coupled with next‐generation companion diagnostics are emerging as solutions to the increasingly recognized issue of molecular heterogeneity. A paradigm shift from ‘one‐size‐fits‐all’ and ‘à la carte’ biomarker diagnostics and patient selection to next‐generation clinical trial designs may allow us to break through the clinical benefit plateau experienced recently with molecularly targeted therapies. Immune therapy holds great promise in the upcoming years as another layer of therapeutics in the arsenal. Regulatory oversight should continue to reasonably assess novel companion diagnostics and next‐generation clinical trial designs, and provide guidance and assurance for regulatory approval to encourage and stimulate innovative approaches, such as testing ‘personalized treatment algorithms’ within the Expansion Platform Type II Design.

## Conflicts of interest

None.

## References

[mol2201595967-bib-0001] Abrams, J. , Conley, B. , Mooney, M. , 2014 National cancer institute's precision medicine initiatives for the new national clinical trials network. Am. Soc. Clin. Oncol. Educ. Book. 71–76. 24857062 10.14694/EdBook_AM.2014.34.71

[mol2201595967-bib-0002] Alix-Panabieres, C. , Pantel, K. , 2014 Challenges in circulating tumour cell research. Nat. Rev. Cancer. 14, 623–631. 25154812 10.1038/nrc3820

[mol2201595967-bib-0003] Arena, V. , Pennacchia, I. , Vecchio, F.M. , Carbone, A. , 2013 HER-2 intratumoral heterogeneity. Mod. Pathol. 26, 607–609. 23542522 10.1038/modpathol.2012.147

[mol2201595967-bib-0004] Baines, A.T. , Xu, D. , Der, C.J. , 2011 Inhibition of Ras for cancer treatment: the search continues. Future Med. Chem. 3, 1787–1808. 22004085 10.4155/fmc.11.121 PMC3347641

[mol2201595967-bib-0005] Bang, Y.J. , Van Cutsem, E. , Feyereislova, A. , 2010 Trastuzumab in combination with chemotherapy versus chemotherapy alone for treatment of HER2-positive advanced gastric or gastro-oesophageal junction cancer (ToGA): a phase 3, open-label, randomised controlled trial. Lancet. 376, 687–697. 20728210 10.1016/S0140-6736(10)61121-X

[mol2201595967-bib-0006] Bang, Y.J. , Im, S.A. , Lee, K.W. , 2013 Olaparib plus paclitaxel in patients with recurrent or metastatic gastric cancer: a randomized, double-blind phase II study. J. Clin. Oncol. 31, Abstr 4013 10.1200/JCO.2014.60.0320 26282658

[mol2201595967-bib-0007] Barker, A.D. , Sigman, C.C. , Kelloff, G.J. , Hylton, N.M. , Berry, D.A. , Esserman, L.J. , 2009 I-SPY 2: an adaptive breast cancer trial design in the setting of neoadjuvant chemotherapy. Clin. Pharmacol. Ther. 86, 97–100. 19440188 10.1038/clpt.2009.68

[mol2201595967-bib-0008] Bedard, P.L. , Hansen, A.R. , Ratain, M.J. , Siu, L.L. , 2013 Tumour heterogeneity in the clinic. Nature. 501, 355–364. 24048068 10.1038/nature12627 PMC5224525

[mol2201595967-bib-0009] Bellou, S. , Pentheroudakis, G. , Murphy, C. , Fotsis, T. , 2013 Anti-angiogenesis in cancer therapy: Hercules and hydra. Cancer Lett. 338, 219–228. 23707856 10.1016/j.canlet.2013.05.015

[mol2201595967-bib-0010] Bianco, R. , Melisi, D. , Ciardiello, F. , Tortora, G. , 2006 Key cancer cell signal transduction pathways as therapeutic targets. Eur. J. Cancer. 42, 290–294. 16376541 10.1016/j.ejca.2005.07.034

[mol2201595967-bib-0011] Blumenschein, G.R. , Saintigny, P. , Liu, S. , 2013 Comprehensive biomarker analysis and final efficacy results of sorafenib in the BATTLE trial. Clin. Cancer Res. 19, 6967–6975. 24166906 10.1158/1078-0432.CCR-12-1818 PMC3905243

[mol2201595967-bib-0012] Catenacci, D.V. , Henderson, L. , Xiao, S.Y. , 2011 Durable complete response of metastatic gastric cancer with anti-Met therapy followed by resistance at recurrence. Cancer Discov. 1, 573–579. 22389872 10.1158/2159-8290.CD-11-0175 PMC3289149

[mol2201595967-bib-0013] Catenacci, D.V. , Cervantes, G. , Yala, S. , 2011 RON (MST1R) is a novel prognostic marker and therapeutic target for gastroesophageal adenocarcinoma. Cancer Biol. Ther. 12, 10.4161/cbt.12.1.15747 PMC3149873 21543897

[mol2201595967-bib-0014] Catenacci, D.V. , Kozloff, M. , Kindler, H.L. , Polite, B. , 2011 Personalized colon cancer care in 2010. Semin. Oncol. 38, 284–308. 21421118 10.1053/j.seminoncol.2011.01.001 PMC3065981

[mol2201595967-bib-0015] Catenacci, D. , Henderson, L. , Liao, W. , Burrows, J. , Hembrough, T. , 2013 KRAS gene amplification defines a distinct molecular subgroup of gastroesophageal adenocarcinoma that may benefit from combined anti-MET/AKT therapy. Cancer Res. Abstr 141239

[mol2201595967-bib-0016] Catenacci, D. , Polite, B. , Henderson, L. , 2014 Towards personalized treatment for gastroesophageal adenocarcinoma (GEC): strategies to address tumor heterogeneity – PANGEA. J. Clin. Oncol. GI ASCO 2014, Abstr 60

[mol2201595967-bib-0017] Catenacci, D.V. , Liao, W.L. , Thyparambil, S. , 2014 Absolute quantitation of met using mass spectrometry for clinical application: assay precision, stability, and correlation with MET gene amplification in FFPE tumor tissue. PLoS One. 9, e100586 24983965 10.1371/journal.pone.0100586 PMC4077664

[mol2201595967-bib-0018] Catenacci, D.V. , Amico, A.L. , Nielsen, S.M. , 2014 Tumor genome analysis includes germline genome: are we ready for surprises?. Int. J. Cancer. 10.1002/ijc.29128 PMC4303936 25123297

[mol2201595967-bib-0019] Cepero, V. , Sierra, J.R. , Corso, S. , 2010 MET and KRAS gene amplification mediates acquired resistance to MET tyrosine kinase inhibitors. Cancer Res. 70, 7580–7590. 20841479 10.1158/0008-5472.CAN-10-0436

[mol2201595967-bib-0020] Chapman, P.B. , Hauschild, A. , Robert, C. , 2011 Improved survival with vemurafenib in melanoma with BRAF V600E mutation. N. Engl. J. Med. 364, 2507–2516. 21639808 10.1056/NEJMoa1103782 PMC3549296

[mol2201595967-bib-0021] Cohen, D.J. , Christos, P.J. , Kindler, H.L. , 2013 Vismodegib (V), a hedgehog (HH) pathway inhibitor, combined with FOLFOX for first-line therapy of patients (pts) with advanced gastric and gastroesophageal (GEJ) carcinoma: a New York Cancer Consortium led phase II randomized study. J. Clin. Oncol. 31, Abstr 4011

[mol2201595967-bib-0022] Collins, F.S. , Hamburg, M.A. , 2013 First FDA authorization for next-generation sequencer. N. Engl. J. Med. 369, 2369–2371. 24251383 10.1056/NEJMp1314561 PMC5101955

[mol2201595967-bib-0023] 2014. Comprehensive molecular characterization of gastric adenocarcinoma. Nature 513, 202–209. 10.1038/nature13480 PMC4170219 25079317

[mol2201595967-bib-0024] Corso, S. , Giordano, S. , 2013 Cell-autonomous and non-cell-autonomous mechanisms of HGF/MET-driven resistance to targeted therapies: from basic research to a clinical perspective. Cancer Discov. 3, 978–992. 23901039 10.1158/2159-8290.CD-13-0040

[mol2201595967-bib-0025] Dancey, J.E. , Dobbin, K.K. , Groshen, S. , 2010 Guidelines for the development and incorporation of biomarker studies in early clinical trials of novel agents. Clin. Cancer Res. 16, 1745–1755. 20215558 10.1158/1078-0432.CCR-09-2167

[mol2201595967-bib-0026] Dancey, J.E. , Bedard, P.L. , Onetto, N. , Hudson, T.J. , 2012 The genetic basis for cancer treatment decisions. Cell. 148, 409–420. 22304912 10.1016/j.cell.2012.01.014

[mol2201595967-bib-0027] De Roock, W. , Claes, B. , Bernasconi, D. , 2010 Effects of KRAS, BRAF, NRAS, and PIK3CA mutations on the efficacy of cetuximab plus chemotherapy in chemotherapy-refractory metastatic colorectal cancer: a retrospective consortium analysis. Lancet Oncol. 11, 753–762. 20619739 10.1016/S1470-2045(10)70130-3

[mol2201595967-bib-0028] Demetri, G.D. , von Mehren, M. , Blanke, C.D. , 2002 Efficacy and safety of imatinib mesylate in advanced gastrointestinal stromal tumors. N. Engl. J. Med. 347, 472–480. 12181401 10.1056/NEJMoa020461

[mol2201595967-bib-0029] Deng, N. , Goh, L.K. , Wang, H. , 2012 A comprehensive survey of genomic alterations in gastric cancer reveals systematic patterns of molecular exclusivity and co-occurrence among distinct therapeutic targets. Gut. 61, 673–684. 22315472 10.1136/gutjnl-2011-301839 PMC3322587

[mol2201595967-bib-0030] Deng, Y. , Wang, C.C. , Choy, K.W. , 2014 Therapeutic potentials of gene silencing by RNA interference: principles, challenges, and new strategies. Gene. 538, 217–227. 24406620 10.1016/j.gene.2013.12.019

[mol2201595967-bib-0031] Devaud, C. , John, L.B. , Westwood, J.A. , Darcy, P.K. , Kershaw, M.H. , 2013 Immune modulation of the tumor microenvironment for enhancing cancer immunotherapy. Oncoimmunology. 2, e25961 24083084 10.4161/onci.25961 PMC3782527

[mol2201595967-bib-0032] Diaz, L.A. , Bardelli, A. , 2014 Liquid biopsies: genotyping circulating tumor DNA. J. Clin. Oncol. 32, 579–586. 24449238 10.1200/JCO.2012.45.2011 PMC4820760

[mol2201595967-bib-0033] Douillard, J.Y. , Rong, A. , Sidhu, R. , 2013 RAS mutations in colorectal cancer. N. Engl. J. Med. 369, 2159–2160. 10.1056/NEJMc1312697 24283232

[mol2201595967-bib-0034] Douillard, J.Y. , Oliner, K.S. , Siena, S. , 2013 Panitumumab-FOLFOX4 treatment and RAS mutations in colorectal cancer. N. Engl. J. Med. 369, 1023–1034. 24024839 10.1056/NEJMoa1305275

[mol2201595967-bib-0035] Dowlati, A. , Haaga, J. , Remick, S.C. , 2001 Sequential tumor biopsies in early phase clinical trials of anticancer agents for pharmacodynamic evaluation. Clin. Cancer Res. 7, 2971–2976. 11595684

[mol2201595967-bib-0036] Druker, B.J. , Guilhot, F. , O'Brien, S.G. , 2006 Five-year follow-up of patients receiving imatinib for chronic myeloid leukemia. N. Engl. J. Med. 355, 2408–2417. 17151364 10.1056/NEJMoa062867

[mol2201595967-bib-0037] Dulak, A.M. , Schumacher, S.E. , van Lieshout, J. , 2012 Gastrointestinal adenocarcinomas of the esophagus, stomach, and colon exhibit distinct patterns of genome instability and oncogenesis. Cancer Res. 72, 4383–4393. 22751462 10.1158/0008-5472.CAN-11-3893 PMC3432726

[mol2201595967-bib-0038] Engelman, J.A. , Zejnullahu, K. , Mitsudomi, T. , 2007 MET amplification leads to gefitinib resistance in lung cancer by activating ERBB3 signaling. Science. 316, 1039–1043. 17463250 10.1126/science.1141478

[mol2201595967-bib-0039] ESMO , 2014 Results from the CALGB/SWOG 80405 and FIRE-3 (AIO KRK-0306) studies in all RAS wild type population. 2014 (accessed at: http://www.esmo.org/Oncology-News/Results-From-the-CALGB-SWOG-80405-and-FIRE-3-AIO-KRK-0306-Studies-In-All-RAS-Wild-Type-Population.http://www.esmo.org/Oncology-News/Results-From-the-CALGB-SWOG-80405-and-FIRE-3-AIO-KRK-0306-Studies-In-All-RAS-Wild-Type-Population

[mol2201595967-bib-0040] Faivre, S. , Djelloul, S. , Raymond, E. , 2006 New paradigms in anticancer therapy: targeting multiple signaling pathways with kinase inhibitors. Semin. Oncol. 33, 407–420. 16890796 10.1053/j.seminoncol.2006.04.005

[mol2201595967-bib-0041] Fauvel, B. , Yasri, A. , 2014 Antibodies directed against receptor tyrosine kinases: current and future strategies to fight cancer. MAbs. 6, 838–851. 24859229 10.4161/mabs.29089 PMC4171019

[mol2201595967-bib-0042] Fearon, E.R. , Vogelstein, B. , 1990 A genetic model for colorectal tumorigenesis. Cell. 61, 759–767. 2188735 10.1016/0092-8674(90)90186-i

[mol2201595967-bib-0043] Fedele, C. , Tothill, R.W. , McArthur, G.A. , 2014 Navigating the challenge of tumor heterogeneity in cancer therapy. Cancer Discov. 4, 146–148. 24501303 10.1158/2159-8290.CD-13-1042

[mol2201595967-bib-0044] Flaherty, K.T. , Puzanov, I. , Kim, K.B. , 2010 Inhibition of mutated, activated BRAF in metastatic melanoma. N. Engl. J. Med. 363, 809–819. 20818844 10.1056/NEJMoa1002011 PMC3724529

[mol2201595967-bib-0045] Frampton, G.M. , Fichtenholtz, A. , Otto, G.A. , 2013 Development and validation of a clinical cancer genomic profiling test based on massively parallel DNA sequencing. Nat. Biotechnol. 31, 1023–1031. 24142049 10.1038/nbt.2696 PMC5710001

[mol2201595967-bib-0046] Freidlin, B. , Korn, E.L. , 2014 Biomarker enrichment strategies: matching trial design to biomarker credentials. Nat. Rev. Clin. Oncol. 11, 81–90. 24281059 10.1038/nrclinonc.2013.218

[mol2201595967-bib-0047] Freidlin, B. , McShane, L.M. , Korn, E.L. , 2010 Randomized clinical trials with biomarkers: design issues. J. Natl. Cancer Inst. 102, 152–160. 20075367 10.1093/jnci/djp477 PMC2911042

[mol2201595967-bib-0048] Fuchs, C.S. , Tomasek, J. , Yong, C.J. , 2014 Ramucirumab monotherapy for previously treated advanced gastric or gastro-oesophageal junction adenocarcinoma (REGARD): an international, randomised, multicentre, placebo-controlled, phase 3 trial. Lancet. 383, 31–39. 24094768 10.1016/S0140-6736(13)61719-5

[mol2201595967-bib-0049] Garraway, L.A. , Verweij, J. , Ballman, K.V. , 2013 Precision oncology: an overview. J. Clin. Oncol. 31, 1803–1805. 23589545 10.1200/JCO.2013.49.4799

[mol2201595967-bib-0050] Gerlinger, M. , Rowan, A.J. , Horswell, S. , 2012 Intratumor heterogeneity and branched evolution revealed by multiregion sequencing. N. Engl. J. Med. 366, 883–892. 22397650 10.1056/NEJMoa1113205 PMC4878653

[mol2201595967-bib-0051] Gimbrone, M.A. , Leapman, S.B. , Cotran, R.S. , Folkman, J. , 1972 Tumor dormancy in vivo by prevention of neovascularization. J. Exp. Med. 136, 261–276. 5043412 10.1084/jem.136.2.261 PMC2139203

[mol2201595967-bib-0052] Gomez-Martin, C. , Plaza, J.C. , Pazo-Cid, R. , 2013 Level of HER2 gene amplification predicts response and overall survival in HER2-positive advanced gastric cancer treated with trastuzumab. J. Clin. Oncol. 31, 4445–4452. 24127447 10.1200/JCO.2013.48.9070

[mol2201595967-bib-0053] Griffin, J. , 2001 The biology of signal transduction inhibition: basic science to novel therapies. Semin. Oncol. 28, 3–8. 11740801

[mol2201595967-bib-0054] Grothey, A. , Sugrue, M.M. , Purdie, D.M. , 2008 Bevacizumab beyond first progression is associated with prolonged overall survival in metastatic colorectal cancer: results from a large observational cohort study (BRiTE). J. Clin. Oncol. 26, 5326–5334. 18854571 10.1200/JCO.2008.16.3212

[mol2201595967-bib-0055] Harris, A.L. , Fox, S. , Bicknell, R. , 1994 Gene therapy through signal transduction pathways and angiogenic growth factors as therapeutic targets in breast cancer. Cancer. 74, 1021–1025. 8039135 10.1002/1097-0142(19940801)74:3+<1021::aid-cncr2820741508>3.0.co;2-1

[mol2201595967-bib-0056] Hecht, J.R. , Bang, Y.J. , Qin, S. , 2013 Lapatinib in combination with capecitabine plus oxaliplatin (CapeOx) in HER2-positive advanced or metastatic gastric, esophgael, or gastroesophageal adenocarcinoma (AC): the TRIO-013/LOGiC Trial. J. Clin. Oncol. 31, abstr LBA4001 10.1200/JCO.2015.62.6598 26628478

[mol2201595967-bib-0057] Hembrough, T. , Liao, W. , Henderson, L. , Xu, P. , Burrows, J. , Catenacci, D. , 2012 Development of a quantitative gastroesophageal cancer selected reaction monitoring mass spectrometric multi-plex assay for use in FFPE tumor tissues. 24th EORTC-NCI-AACR Symposium Dublin, Ireland November 6-9, 2012. (Abstr 561)

[mol2201595967-bib-0058] Hirsch, F.R. , Bunn, P.A. , Herbst, R.S. , 2014 “Companion diagnostics”: has their time come and gone?. Clin. Cancer Res. 20, 4422–4424. 25059519 10.1158/1078-0432.CCR-14-0932 PMC4155019

[mol2201595967-bib-0059] Holbrook, J.D. , Parker, J.S. , Gallagher, K.T. , 2011 Deep sequencing of gastric carcinoma reveals somatic mutations relevant to personalized medicine. J. Transl. Med. 9, 119 21781349 10.1186/1479-5876-9-119 PMC3152520

[mol2201595967-bib-0060] Hull, D.L. , 2005 Deconstructing Darwin: evolutionary theory in context. J. Hist. Biol. 38, 137–152. 25214421 10.1007/s10739-004-6514-1

[mol2201595967-bib-0061] Islam, M.F. , Hoque, M.M. , Banik, R.S. , 2013 Comparative analysis of differential network modularity in tissue specific normal and cancer protein interaction networks. J. Clin. Bioinforma. 3, 19 24093757 10.1186/2043-9113-3-19 PMC3852839

[mol2201595967-bib-0062] Iveson, T. , Donehower, R.C. , Davidenko, I. , 2014 Rilotumumab in combination with epirubicin, cisplatin, and capecitabine as first-line treatment for gastric or oesophagogastric junction adenocarcinoma: an open-label, dose de-escalation phase 1b study and a double-blind, randomised phase 2 study. Lancet Oncol. 10.1016/S1470-2045(14)70023-3 24965569

[mol2201595967-bib-0063] Joensuu, H. , 2008 Systemic chemotherapy for cancer: from weapon to treatment. Lancet Oncol. 9, 304 18308256 10.1016/S1470-2045(08)70075-5

[mol2201595967-bib-0064] June, C.H. , Maus, M.V. , Plesa, G. , 2014 Engineered T cells for cancer therapy. Cancer Immunol. Immunother. 63, 969–975. 24943274 10.1007/s00262-014-1568-1 PMC4142345

[mol2201595967-bib-0065] Kaelin, W.G. , 2005 The concept of synthetic lethality in the context of anticancer therapy. Nat. Rev. Cancer. 5, 689–698. 16110319 10.1038/nrc1691

[mol2201595967-bib-0066] Kakarla, S. , Song, X.T. , Gottschalk, S. , 2012 Cancer-associated fibroblasts as targets for immunotherapy. Immunotherapy. 4, 1129–1138. 23194363 10.2217/imt.12.112 PMC3568630

[mol2201595967-bib-0067] Kaplan, R. , Maughan, T. , Crook, A. , 2013 Evaluating many treatments and biomarkers in oncology: a new design. J. Clin. Oncol. 31, 4562–4568. 24248692 10.1200/JCO.2013.50.7905 PMC4394353

[mol2201595967-bib-0068] Karaman, M.W. , Herrgard, S. , Treiber, D.K. , 2008 A quantitative analysis of kinase inhibitor selectivity. Nat. Biotechnol. 26, 127–132. 18183025 10.1038/nbt1358

[mol2201595967-bib-0069] Keenan, B.P. , Jaffee, E.M. , Armstrong, T.D. , 2013 Tumor immunology: multidisciplinary science driving basic and clinical advances. Cancer Immunol. Res. 1, 16–23. 24409447 10.1158/2326-6066.CIR-13-0011 PMC3882090

[mol2201595967-bib-0070] Khoo, B.L. , Warkiani, M.E. , Tan, D.S. , 2014 Clinical validation of an ultra high-throughput spiral microfluidics for the detection and enrichment of viable circulating tumor cells. PLoS One. 9, e99409 24999991 10.1371/journal.pone.0099409 PMC4085042

[mol2201595967-bib-0071] Khoury, J.D. , Catenacci, D.V. , 2014 Next-generation companion diagnostics: promises, challenges, and solutions. Arch. Pathol. Lab Med. 10.5858/arpa.2014-0063-ED PMC4991626 25166874

[mol2201595967-bib-0072] Kim, E.S. , Herbst, R.S. , Wistuba , 2011 The BATTLE trial: personalizing therapy for lung cancer. Cancer Discov. 1, 44–53. 22586319 10.1158/2159-8274.CD-10-0010 PMC4211116

[mol2201595967-bib-0073] Kurzrock, R. , Kantarjian, H. , Stewart, D.J. , 2014 A cancer trial scandal and its regulatory backlash. Nat. Biotechnol. 32, 27–31. 24406925 10.1038/nbt.2792

[mol2201595967-bib-0074] Lai, T.L. , Lavori, P.W. , Shih, M.C. , Sikic, B.I. , 2012 Clinical trial designs for testing biomarker-based personalized therapies. Clin. Trials. 9, 141–154. 22397801 10.1177/1740774512437252 PMC4296980

[mol2201595967-bib-0075] Le, D.T. , Jaffee, E.M. , 2013 Harnessing immune responses in the tumor microenvironment: all signals needed. Clin. Cancer Res. 19, 6061–6063. 24097857 10.1158/1078-0432.CCR-13-2424 PMC3839670

[mol2201595967-bib-0076] Leary, A. , Johnston, S.R. , 2007 Small molecule signal transduction inhibitors for the treatment of solid tumors. Cancer Invest. 25, 347–365. 17661211 10.1080/07357900701259694

[mol2201595967-bib-0077] Leary, R.J. , Sausen, M. , Kinde, I. , 2012 Detection of chromosomal alterations in the circulation of cancer patients with whole-genome sequencing. Sci. Transl. Med. 4, 162ra54 10.1126/scitranslmed.3004742 PMC3641759 23197571

[mol2201595967-bib-0078] Lee, H.E. , Park, K.U. , Yoo, S.B. , 2013 Clinical significance of intratumoral HER2 heterogeneity in gastric cancer. Eur. J. Cancer. 49, 1448–1457. 23146959 10.1016/j.ejca.2012.10.018

[mol2201595967-bib-0079] Lee, J.M. , Hays, J.L. , Noonan, A.M. , 2013 Feasibility and safety of sequential research-related tumor core biopsies in clinical trials. Cancer. 119, 1357–1364. 23280317 10.1002/cncr.27916 PMC3604070

[mol2201595967-bib-0080] Lengauer, C. , Diaz, L.A. , Saha, S. , 2005 Cancer drug discovery through collaboration. Nat. Rev. Drug Discov. 4, 375–380. 15864266 10.1038/nrd1722

[mol2201595967-bib-0081] Longo, D.L. , 2012 Tumor heterogeneity and personalized medicine. N. Engl. J. Med. 366, 956–957. 22397658 10.1056/NEJMe1200656

[mol2201595967-bib-0082] Lordick, F. , Kang, Y.K. , Chung, H.C. , 2013 Capecitabine and cisplatin with or without cetuximab for patients with previously untreated advanced gastric cancer (EXPAND): a randomised, open-label phase 3 trial. Lancet Oncol. 14, 490–499. 23594786 10.1016/S1470-2045(13)70102-5

[mol2201595967-bib-0083] Lordick, F. , kang, Y. , Salman, P. , 2013 Clinical outcome according to tumor HER2 status and EGFR expression in advanced gastric cancer from the EXPAND study. J. Clin. Oncol. 31, Suppl. ; abstr 2012

[mol2201595967-bib-0084] Mandrekar, S.J. , Sargent, D.J. , 2011 Design of clinical trials for biomarker research in oncology. Clin. Investig. (Lond). 1, 1629–1636. 10.4155/CLI.11.152 PMC3290127 22389760

[mol2201595967-bib-0085] Mauro, M.J. , O'Dwyer, M. , Heinrich, M.C. , Druker, B.J. , 2002 STI571: a paradigm of new agents for cancer therapeutics. J. Clin. Oncol. 20, 325–334. 11773186 10.1200/JCO.2002.20.1.325

[mol2201595967-bib-0086] Maus, M.V. , Fraietta, J.A. , Levine, B.L. , Kalos, M. , Zhao, Y. , June, C.H. , 2014 Adoptive immunotherapy for cancer or viruses. Annu. Rev. Immunol. 32, 189–225. 24423116 10.1146/annurev-immunol-032713-120136 PMC4533835

[mol2201595967-bib-0087] Melero, I. , Gaudernack, G. , Gerritsen, W. , 2014 Therapeutic vaccines for cancer: an overview of clinical trials. Nat. Rev. Clin. Oncol. 11, 509–524. 25001465 10.1038/nrclinonc.2014.111

[mol2201595967-bib-0088] Mellman, I. , Coukos, G. , Dranoff, G. , 2011 Cancer immunotherapy comes of age. Nature. 480, 480–489. 22193102 10.1038/nature10673 PMC3967235

[mol2201595967-bib-0089] Mendelsohn, J. , 2013 Personalizing oncology: perspectives and prospects. J. Clin. Oncol. 31, 1904–1911. 23589547 10.1200/JCO.2012.45.3605

[mol2201595967-bib-0090] Mueller, M.M. , Fusenig, N.E. , 2004 Friends or foes – bipolar effects of the tumour stroma in cancer. Nat. Rev. Cancer. 4, 839–849. 15516957 10.1038/nrc1477

[mol2201595967-bib-0091] Muro, K. , Bang, Y.J. , Shankaran, V. , 2014 A phase 1b study of Pembrolizumab (Pembro; MK-3475) in patients with advanced gastric Cancer. ESMO. Oral Abstr LBA15 2014

[mol2201595967-bib-0092] Navin, N.E. , Hicks, J. , 2010 Tracing the tumor lineage. Mol. Oncol. 4, 267–283. 20537601 10.1016/j.molonc.2010.04.010 PMC2904844

[mol2201595967-bib-0093] Navin, N. , Krasnitz, A. , Rodgers, L. , 2010 Inferring tumor progression from genomic heterogeneity. Genome Res. 20, 68–80. 19903760 10.1101/gr.099622.109 PMC2798832

[mol2201595967-bib-0094] 2014. Neratinib graduates to I-SPY 3. Cancer Discov. 4, 624. 10.1158/2159-8290.CD-NB2014-055 24891343

[mol2201595967-bib-0095] Nowell, P.C. , 1976 The clonal evolution of tumor cell populations. Science. 194, 23–28. 959840 10.1126/science.959840

[mol2201595967-bib-0096] Ohtsu, A. , Shah, M.A. , Van Cutsem, E. , 2011 Bevacizumab in combination with chemotherapy as first-line therapy in advanced gastric cancer: a randomized, double-blind, placebo-controlled phase III study. J. Clin. Oncol. 29, 3968–3976. 21844504 10.1200/JCO.2011.36.2236

[mol2201595967-bib-0097] Ohtsu, A. , Ajani, J.A. , Bai, Y.X. , 2013 Everolimus for previously treated advanced gastric cancer: results of the randomized, double-blind, phase III GRANITE-1 study. J. Clin. Oncol. 31, 3935–3943. 24043745 10.1200/JCO.2012.48.3552 PMC5950503

[mol2201595967-bib-0098] Olopade, O.I. , 2014 Obituary: Janet Davison Rowley 1925–2013. Cell. 156, 390–391. 24757717 10.1016/j.cell.2014.01.015

[mol2201595967-bib-0099] Overman, M.J. , Modak, J. , Kopetz, S. , 2013 Use of research biopsies in clinical trials: are risks and benefits adequately discussed?. J. Clin. Oncol. 31, 17–22. 23129736 10.1200/JCO.2012.43.1718 PMC5545102

[mol2201595967-bib-0100] Pantel, K. , Alix-Panabieres, C. , 2013 Real-time liquid biopsy in cancer patients: fact or fiction?. Cancer Res. 73, 6384–6388. 24145355 10.1158/0008-5472.CAN-13-2030

[mol2201595967-bib-0101] Parkinson, D.R. , Johnson, B.E. , Sledge, G.W. , 2012 Making personalized cancer medicine a reality: challenges and opportunities in the development of biomarkers and companion diagnostics. Clin. Cancer Res. 18, 619–624. 22298894 10.1158/1078-0432.CCR-11-2017

[mol2201595967-bib-0102] Parmigiani, G. , Boca, S. , Lin, J. , Kinzler, K.W. , Velculescu, V. , Vogelstein, B. , 2009 Design and analysis issues in genome-wide somatic mutation studies of cancer. Genomics. 93, 17–21. 18692126 10.1016/j.ygeno.2008.07.005 PMC2820387

[mol2201595967-bib-0103] Pegram, M. , Slamon, D. , 2000 Biological rationale for HER2/neu (c-erbB2) as a target for monoclonal antibody therapy. Semin. Oncol. 27, 13–19. 11049052

[mol2201595967-bib-0104] Petty, R.D. , Dahle-Smith, A. , Miedzybrodzka, Z. , 2014 Epidermal growth factor receptor copy number gain (EGFR CNG) and response to gefitinib in esophageal cancer (EC): results of biomarker analysis of a phase III trial of gefitinib versus placebo (TRANS-COG). J. Clin. Oncol. 32, 5s, Abstr 4016

[mol2201595967-bib-0105] Poplin, E. , Wasan, H. , Rolfe, L. , 2013 Randomized, multicenter, phase II study of CO-101 versus gemcitabine in patients with metastatic pancreatic ductal adenocarcinoma: including a prospective evaluation of the role of hENT1 in gemcitabine or CO-101 sensitivity. J. Clin. Oncol. 31, 4453–4461. 24220555 10.1200/JCO.2013.51.0826

[mol2201595967-bib-0106] 2014. Positive results for drug combo in I-SPY 2 trial. Cancer Discov. 4 (OF2). 10.1158/2159-8290.CD-NB2013-182 24501314

[mol2201595967-bib-0107] Rowley, J.D. , 1973 Identification of a translocation with quinacrine fluorescence in a patient with acute leukemia. Ann. Genet. 16, 109–112. 4125056

[mol2201595967-bib-0108] Rowley, J.D. , Wolman, S.R. , Horland, A.A. , 1976 Letter: another variant translocation in chronic myelogenous leukemia–revisited. New Engl. J. Med. 295, 900–901. 1066525 10.1056/NEJM197610142951618

[mol2201595967-bib-0109] Sargent, D.J. , Conley, B.A. , Allegra, C. , Collette, L. , 2005 Clinical trial designs for predictive marker validation in cancer treatment trials. J. Clin. Oncol. 23, 2020–2027. 15774793 10.1200/JCO.2005.01.112

[mol2201595967-bib-0110] Satoh, T. , Xu, R.H. , Chung, H.C. , 2014 Lapatinib plus paclitaxel versus paclitaxel alone in the second-line treatment of HER2-amplified advanced gastric cancer in Asian populations: TyTAN-a randomized, phase III study. J. Clin. Oncol. 32, 2039–2049. 24868024 10.1200/JCO.2013.53.6136

[mol2201595967-bib-0111] Schwarzenbach, H. , Nishida, N. , Calin, G.A. , Pantel, K. , 2014 Clinical relevance of circulating cell-free microRNAs in cancer. Nat. Rev. Clin. Oncol. 11, 145–156. 24492836 10.1038/nrclinonc.2014.5

[mol2201595967-bib-0112] Sehdev, A. , Catenacci, D.V. , 2013 Perioperative therapy for locally advanced gastroesophageal cancer: current controversies and consensus of care. J. Hematol. Oncol. 6, 66 24010946 10.1186/1756-8722-6-66 PMC3844370

[mol2201595967-bib-0113] Sehdev, A. , Catenacci, D.V. , 2013 Gastroesophageal cancer: focus on epidemiology, classification, and staging. Discov. Med. 16, 103–111. 23998446

[mol2201595967-bib-0114] Seol, H. , Lee, H.J. , Choi, Y. , 2012 Intratumoral heterogeneity of HER2 gene amplification in breast cancer: its clinicopathological significance. Mod. Pathol. 25, 938–948. 22388760 10.1038/modpathol.2012.36

[mol2201595967-bib-0115] Shimada, H. , 2014 Is “liquid biopsy” useful for assessing HER2 status in gastric cancer?. J. Gastroenterol. 10.1007/s00535-014-0967-6 24825793

[mol2201595967-bib-0116] Shojaei, F. , 2012 Anti-angiogenesis therapy in cancer: current challenges and future perspectives. Cancer Lett. 320, 130–137. 22425960 10.1016/j.canlet.2012.03.008

[mol2201595967-bib-0117] Simon, R. , Maitournam, A. , 2004 Evaluating the efficiency of targeted designs for randomized clinical trials. Clin. Cancer Res. 10, 6759–6763. 15501951 10.1158/1078-0432.CCR-04-0496

[mol2201595967-bib-0118] Simon, R.M. , Paik, S. , Hayes, D.F. , 2009 Use of archived specimens in evaluation of prognostic and predictive biomarkers. J. Natl. Cancer Inst. 101, 1446–1452. 19815849 10.1093/jnci/djp335 PMC2782246

[mol2201595967-bib-0119] Slamon, D.J. , deKernion, J.B. , Verma, I.M. , Cline, M.J. , 1984 Expression of cellular oncogenes in human malignancies. Science. 224, 256–262. 6538699 10.1126/science.6538699

[mol2201595967-bib-0120] Slamon, D.J. , Clark, G.M. , Wong, S.G. , Levin, W.J. , Ullrich, A. , McGuire, W.L. , 1987 Human breast cancer: correlation of relapse and survival with amplification of the HER-2/neu oncogene. Science. 235, 177–182. 3798106 10.1126/science.3798106

[mol2201595967-bib-0121] Slamon, D.J. , Leyland-Jones, B. , Shak, S. , 2001 Use of chemotherapy plus a monoclonal antibody against HER2 for metastatic breast cancer that overexpresses HER2. N. Engl. J. Med. 344, 783–792. 11248153 10.1056/NEJM200103153441101

[mol2201595967-bib-0122] Smolen, G.A. , Sordella, R. , Muir, B. , 2006 Amplification of MET may identify a subset of cancers with extreme sensitivity to the selective tyrosine kinase inhibitor PHA-665752. Proc. Natl. Acad. Sci. U S A. 103, 2316–2321. 16461907 10.1073/pnas.0508776103 PMC1413705

[mol2201595967-bib-0123] Speicher, M.R. , Pantel, K. , 2014 Tumor signatures in the blood. Nat. Biotechnol. 32, 441–443. 24811515 10.1038/nbt.2897

[mol2201595967-bib-0124] Stricker, T. , Catenacci, D.V. , Seiwert, T.Y. , 2011 Molecular profiling of cancer-the future of personalized cancer medicine: a primer on cancer biology and the tools necessary to bring molecular testing to the clinic. Semin. Oncol. 38, 173–185. 21421108 10.1053/j.seminoncol.2011.01.013

[mol2201595967-bib-0125] Sullivan, R.J. , Lorusso, P.M. , Flaherty, K.T. , 2013 The intersection of immune-directed and molecularly targeted therapy in advanced melanoma: where we have been, are, and will be. Clin. Cancer Res. 19, 5283–5291. 24089441 10.1158/1078-0432.CCR-13-2151 PMC4100326

[mol2201595967-bib-0126] Swanton, C. , 2012 Intratumor heterogeneity: evolution through space and time. Cancer Res. 72, 4875–4882. 23002210 10.1158/0008-5472.CAN-12-2217 PMC3712191

[mol2201595967-bib-0127] Tsao, A.S. , Liu, S. , Lee, J.J. , 2013 Clinical and biomarker outcomes of the phase II vandetanib study from the BATTLE trial. J. Thorac. Oncol. 8, 658–661. 23584298 10.1097/JTO.0b013e31828d08ae PMC5118909

[mol2201595967-bib-0128] Turke, A.B. , Zejnullahu, K. , Wu, Y.L. , 2010 Preexistence and clonal selection of MET amplification in EGFR mutant NSCLC. Cancer Cell. 17, 77–88. 20129249 10.1016/j.ccr.2009.11.022 PMC2980857

[mol2201595967-bib-0129] Vakiani, E. , Janakiraman, M. , Shen, R. , 2012 Comparative genomic analysis of primary versus metastatic colorectal carcinomas. J. Clin. Oncol. 30, 2956–2962. 22665543 10.1200/JCO.2011.38.2994 PMC3417049

[mol2201595967-bib-0130] Verma, S. , Miles, D. , Gianni, L. , 2012 Trastuzumab emtansine for HER2-positive advanced breast cancer. N. Engl. J. Med. 367, 1783–1791. 23020162 10.1056/NEJMoa1209124 PMC5125250

[mol2201595967-bib-0131] Videira, M. , Arranja, A. , Rafael, D. , Gaspar, R. , 2014 Preclinical development of siRNA therapeutics: towards the match between fundamental science and engineered systems. Nanomedicine. 10, 689–702. 24333589 10.1016/j.nano.2013.11.018

[mol2201595967-bib-0132] Vignot, S. , Frampton, G.M. , Soria, J.C. , 2013 Next-generation sequencing reveals high concordance of recurrent somatic alterations between primary tumor and metastases from patients with non-small-cell lung cancer. J. Clin. Oncol. 31, 2167–2172. 23630207 10.1200/JCO.2012.47.7737

[mol2201595967-bib-0133] Vogelstein, B. , Papadopoulos, N. , Velculescu, V.E. , Zhou, S. , Diaz, L.A. , Kinzler, K.W. , 2013 Cancer genome landscapes. Science. 339, 1546–1558. 23539594 10.1126/science.1235122 PMC3749880

[mol2201595967-bib-0134] von Minckwitz, G. , du Bois, A. , Schmidt, M. , 2009 Trastuzumab beyond progression in human epidermal growth factor receptor 2-positive advanced breast cancer: a german breast group 26/breast international group 03-05 study. J. Clin. Oncol. 27, 1999–2006. 19289619 10.1200/JCO.2008.19.6618

[mol2201595967-bib-0135] Waddell, T. , Chau, I. , Cunningham, D. , 2013 Epirubicin, oxaliplatin, and capecitabine with or without panitumumab for patients with previously untreated advanced oesophagogastric cancer (REAL3): a randomised, open-label phase 3 trial. Lancet Oncol. 14, 481–489. 23594787 10.1016/S1470-2045(13)70096-2 PMC3669518

[mol2201595967-bib-0136] Wang, K. , Kan, J. , Yuen, S.T. , 2011 Exome sequencing identifies frequent mutation of ARID1A in molecular subtypes of gastric cancer. Nat. Genet. 43, 1219–1223. 22037554 10.1038/ng.982

[mol2201595967-bib-0137] Wang, S.J. , Hung, H.M. , O'Neill, R. , 2012 Paradigms for adaptive statistical information designs: practical experiences and strategies. Stat. Med. 31, 3011–3023. 22927234 10.1002/sim.5410

[mol2201595967-bib-0138] Warth, A. , Muley, T. , Herpel, E. , 2012 Large-scale comparative analyses of immunomarkers for diagnostic subtyping of non-small-cell lung cancer biopsies. Histopathology. 61, 1017–1025. 22882703 10.1111/j.1365-2559.2012.04308.x

[mol2201595967-bib-0139] Weinstein, I.B. , 2002 Cancer. Addiction to oncogenes–the Achilles heal of cancer. Science. 297, 63–64. 12098689 10.1126/science.1073096

[mol2201595967-bib-0140] Weinstein, I.B. , Joe, A. , 2008 Oncogene addiction. Cancer Res. 68, 3077–3080. discussion 80 18451130 10.1158/0008-5472.CAN-07-3293

[mol2201595967-bib-0141] Wilke, H. , Muro, K. , Van Cutsem, E. , 2014 Ramucirumab plus paclitaxel versus placebo plus paclitaxel in patients with previously treated advanced gastric or gastro-oesophageal junction adenocarcinoma (RAINBOW): a double-blind, randomised phase 3 trial. Lancet Oncol. 10.1016/S1470-2045(14)70420-6 25240821

[mol2201595967-bib-0142] Wilson, T.R. , Fridlyand, J. , Yan, Y. , 2012 Widespread potential for growth-factor-driven resistance to anticancer kinase inhibitors. Nature. 487, 505–509. 22763448 10.1038/nature11249 PMC3724525

[mol2201595967-bib-0143] Yan, R. , Hallam, A. , Stockley, P.G. , Boyes, J. , 2014 Oncogene dependency and the potential of targeted RNAi-based anti-cancer therapy. Biochem. J. 461, 1–13. 24927119 10.1042/BJ20140173

[mol2201595967-bib-0144] Yap, T.A. , Gerlinger, M. , Futreal, P.A. , Pusztai, L. , Swanton, C. , 2012 Intratumor heterogeneity: seeing the wood for the trees. Sci. Transl. Med. 4, 127ps10 10.1126/scitranslmed.3003854 22461637

[mol2201595967-bib-0145] Yap, T.A. , Omlin, A. , de Bono, J.S. , 2013 Development of therapeutic combinations targeting major cancer signaling pathways. J. Clin. Oncol. 31, 1592–1605. 23509311 10.1200/JCO.2011.37.6418

[mol2201595967-bib-0146] Yoon, H.H. , Shi, Q. , Sukov, W.R. , 2012 Adverse prognostic impact of intratumor heterogeneous HER2 gene amplification in patients with esophageal adenocarcinoma. Journal of clinical oncology. official J. Am. Soc. Clin. Oncol. 30, 3932–3938. 10.1200/JCO.2012.43.1890 PMC3675687 22987085

[mol2201595967-bib-0147] Zang, Z.J. , Cutcutache, I. , Poon, S.L. , 2012 Exome sequencing of gastric adenocarcinoma identifies recurrent somatic mutations in cell adhesion and chromatin remodeling genes. Nat. Genet. 44, 570–574. 22484628 10.1038/ng.2246

[mol2201595967-bib-0148] Zhang, L. , Yang, J. , Cai, J. , 2013 A subset of gastric cancers with EGFR amplification and overexpression respond to cetuximab therapy. Sci. Rep. 3, 2992 24141978 10.1038/srep02992 PMC3801116

[mol2201595967-bib-0149] Zhou, C. , Wu, Y.L. , Chen, G. , 2011 Erlotinib versus chemotherapy as first-line treatment for patients with advanced EGFR mutation-positive non-small-cell lung cancer (OPTIMAL, CTONG-0802): a multicentre, open-label, randomised, phase 3 study. Lancet Oncol. 12, 735–742. 21783417 10.1016/S1470-2045(11)70184-X

[mol2201595967-bib-0150] Zitvogel, L. , Tesniere, A. , Kroemer, G. , 2006 Cancer despite immunosurveillance: immunoselection and immunosubversion. Nat. Rev. Immunol. 6, 715–727. 16977338 10.1038/nri1936

